# Biomechanically informed improvements to the brain–skull interface in an atlas-based brain model

**DOI:** 10.3389/fbioe.2026.1881544

**Published:** 2026-10-29

**Authors:** Logan E. Miller, Cole D. Smith, Bharath Koya, Stephen B. Tatter, Alexander K. Powers, Jillian E. Urban, Joel D. Stitzel

**Affiliations:** 1 Department of Biomedical Engineering, Wake Forest University School of Medicine, Winston-Salem, NC, United States; 2 Department of Neurosurgery, Wake Forest University School of Medicine, Winston-Salem, NC, United States; 3 Covenant Spine and Neurology, Winston-Salem, NC, United States

**Keywords:** biofidelity, brain model, CORA, finite element model, validation

## Abstract

**Introduction:**

Finite element (FE) models are powerful tools for injury analysis and prediction. Their performance should be assessed with a standardized system to validate their use before employing them for injury prediction. It is necessary to develop an FE model that is able to reproduce the local response of a wide range of experimental conditions for the intended use. Because there is no standard for quantifying model performance, different validated brain models can produce varying regional pressure and strain responses, which motivates the use of a standard validation protocol, as used in the current study.

**Methods:**

In the current study, biofidelity improvements were made to an existing atlas-based brain FE model developed from the International Consortium for Brain Mapping (ICBM) brain atlas. The brain-skull interface was enhanced by decoupling the current skull and cerebrospinal fluid (CSF) parts and implementing a smooth skull geometry and a sliding with no separation contact at the skull interface. This new model is an enhancement of the atlas-based brain model (ABM) and will be referred to as ABMv4. The new, decoupled model was tested in various experimental impacts and its performance against validation data assessed using CORA (CORrelation and Analysis), an objective rating method.

**Results:**

A smooth surface at the skull interface was implemented to create the ABMv4. The ABMv4 was then validated against 23 experimental cadaver impacts - sixteen ICP tests, nine strain tests, and seventeen brain displacement tests. The improved model achieved CORA ratings of 0.733, 0.563, 0.846, and 0.605 for ICP, brain strain, revised brain strain, and brain displacement validation tests, respectively.

**Discussion:**

The ABMv4 performs well in all types of validation tests and achieves a good biofidelity rating. Additionally, the ABMv4 results in increased relative brain-skull motion (average of 0.54 mm for ABMv2 compared to average of 1.06 mm for ABMv4) and reduced brain strain on the surface of the model, with the surface strain being on average 61.7% of the surface strain value predicted for the ABMv2.

## 1 Introduction

Finite element (FE) brain models have been developed and extensively investigated as a powerful method to study brain injuries (Chafi et al., 2009; Tse et al., 2014; Dixit and Liu, 2017; Zhao et al., 2018; Giudice et al., 2019; Ji et al., 2022; Raouf et al., 2025). Brain FE models have been used and in development since the 1990s and use a range of anatomic detail, material properties, and modeling approaches (Zhang et al., 2001; Horgan and Gilchrist, 2003; Belingardi et al., 2005; Kleiven, 2007; Mao et al., 2013; Fernandes et al., 2018; Gomes et al., 2024). Once properly validated, these FE models can be used to simulate and predict brain response and reconstruct events where brain injury occurs under given impact conditions (Yang and King, 2011; Giordano and Kleiven, 2016; Zhou et al., 2019c). There are several sets of experimental tests that are commonly used to validate FE brain models. These include intracranial pressure (ICP) experiments, brain strain, and relative displacement tests (Nahum et al., 1977; Trosseille et al., 1992; Hardy et al., 2007).

Advanced medical imaging techniques such as magnetic resonance imaging and computed tomography have allowed accurate representation of the head and brain geometry with sub-millimeter precision (Giudice et al., 2020; Alshareef et al., 2021; Li et al., 2021); the mechanical response of these complex tissues and their interactions, however, still pose various challenges for human body modelers. Linear viscoelastic and hyper-viscoelastic material models are commonly used to model brain tissue, though a wide range of parameters exist across the literature (Takhounts et al., 2003; Ghajari et al., 2017; Zhao and Ji, 2019; 2020; Giudice et al., 2021; Lyu et al., 2022). Just as important as the material model and parameters selected for the brain tissue are those selected for the cerebrospinal fluid (CSF). The CSF model, as well as the model of the brain-skull interface, are critical to accurately predict brain response to impact since both completely surround the brain and transfer forces from the skull to the brain tissue. Experimental investigation of human brain tissue deformation under impact loading conditions reveals more information about deformation toward the center of the brain *versus* closer to the surface of the brain (Hardy et al., 2007; Jin et al., 2007 inferred that the arachnoid trabeculae are essential to anchor the brain within the skull, limiting relative motion between the brain and skull. Most FE models do not include detailed trabeculae, but model their effect through shear stiffness of the CSF (Khasawneh et al., 2018). Additionally, for numerical stability reasons, CSF is commonly modeled with a reduced bulk modulus value, thus having lower bulk stiffness than in reality (Li et al., 2019).

The brain-skull interface is an important aspect of a brain FE model; allowance of relative motion between the brain and skull is an important but traditionally under-emphasized feature for simulating brain response. The most recent version of the atlas-based brain model (ABMv2) used a voxel-based mesh-generation approach, resulting in all interfaces exhibiting ‘stair-stepped’ features (Miller et al., 2016). This approach and the associated model parameters limit relative motion between the skull and CSF and may under-represent relative motion between the skull and brain and cause overestimates of strain at the surface of the brain. Many models (Zhang et al., 2001; Mao et al., 2013; Iwamoto and Nakahira, 2015; Ghajari et al., 2017; Zhao and Ji, 2020; Giudice et al., 2021) use a tied interface at the skull to represent the brain-skull interface which may be an oversimplification of the biomechanics at this interface.

Validated FE brain models have been used to improve injury prediction capabilities and the interpretation of subconcussive head impact exposure in sport, automotive, and military environments (Ji et al., 2022). The ABM and other brain models have quantified tissue-level response during football (Miller et al., 2019; Milef et al., 2022), soccer (Filben et al., 2021; Barnes-Wood et al., 2024), hockey (Levy et al., 2021), and other sports. These tissue-level predictions have been correlated with imaging changes, traumatic brain injury (TBI) risk, and skull fracture risk. Strain metrics such as cumulative tensile strain and strain rate in non-injured youth football players have been correlated with regional white matter diffusion changes (Holcomb et al., 2021); additionally, other cumulative strain-based measures have been shown to be better discriminators of imaging changes than kinematic-based measures (Miller et al., 2022). Helmeted football impacts have shown that brain strain profiles vary with head impact location and player position which could influence injury risk (Elkin et al., 2019; Karton et al., 2020).

FE models are powerful tools, but their performance should be assessed with a standardized system before using them for injury prediction. It is necessary to develop an FE model that is able to reproduce the local response of a wide range of experimental conditions for the intended use. The set of validation tests should represent the entire range of conceivable impacts across a range of magnitudes. Furthermore, the model’s prediction of ICP, skull-brain relative displacement and brain deformation should be assessed. This is highlighted by the fact that in the past it has been observed that correct pressure prediction by the model does not necessarily mean that the prediction of the displacement and strain is correct (Kleiven and Hardy, 2002). Because there is no standard for quantifying model performance, different validated brain models can produce varying regional pressure and strain responses (Baeck, 2013; Ji et al., 2014). To address this lack of standardization, Giordano and Kleiven (2016) proposed a validation protocol for assessing the biofidelity of FE head models consisting of 40 experimental cases of ICP, brain motion and brain deformation from a total of 75 experimental cases examined (Giordano and Kleiven, 2016).

Motion at the brain-skull interface has been of interest and quantified through various methods for many years, including through the high-speed biplane x-ray system and neutral density targets (NDTs) used in the validation of brain motion. Although Hardy et al. have conducted many experiments using this approach, the brain motion measurements are limited to the locations of the implanted NDTs, which tend to be deep in the brain, as shown in Figure 1C. The NDTs closest to the skull are generally ∼20–40 mm from the skull, while the brain surface is on average 2.1 mm from the skull. More recently, studies have used magnetic resonance imaging (MRI) to quantify movement and deformation of the brain in human volunteers (Ji et al., 2004; Bayly et al., 2005; Feng et al., 2010; Chen et al., 2012). While this approach offers results and measurements of brain motion at all locations in the brain, including at the brain-skull interface, these tests are run at much lower magnitudes than necessary to validate FE models for brain injury prediction since they are conducted with volunteers. A study by Ji et al. (2004) examined cerebellum and brain stem relative motion in slow, controlled drop experiments and noted that during flexion, the cerebellum rotated in the flexion direction, exceeding the skull rotation, but relative brain stem rotations were negligible. With a flexion angle of 54°, the 95% confidence interval for the relative cerebellum rotation was 2.7°–4.3°, and 0.8–1.6 mm for the relative brain stem axial displacement (Ji et al., 2004). A study examining controlled mild acceleration occipital impacts on human volunteers noted that rotation of the brain relative to the skull can occur even during linear input. Along with this rotation, strain develops as part of the brain compresses against the frontal region of the skull and stretching occurs as the brain is pulled away from the skull in the posterior region. Strains of 0.02–0.05 were typical during these events (0.05 strain corresponds roughly to a 5% change in the dimension of a local tissue element) (Bayly et al., 2005). Feng et al. (2010) reports relative displacement between the brain and the skull *in vivo* using tagged magnetic resonance imaging and digital image analysis under mild acceleration events in volunteers (Feng et al., 2010). For these mild events, characterized by linear decelerations near 1.5 g and angular accelerations of 120–140 rad/s^2^, relative brain–skull displacements of 2–3 mm are typical. In a study of mild impact head drop experiment using tagged MRI and harmonic phase imaging analysis, it was found that the maximum deformations occur within a few milliseconds following the impact, which is during the first oscillation of the brain within the skull, with the maximum displacements of 2–3 mm and the maximum strains of 5–10% (Chen et al., 2012). These various studies examining and reporting relative motion provide valuable insight into brain deformation under a range of conditions, but do not specifically capture the relative displacement at the brain skull interface.

**FIGURE 1:**
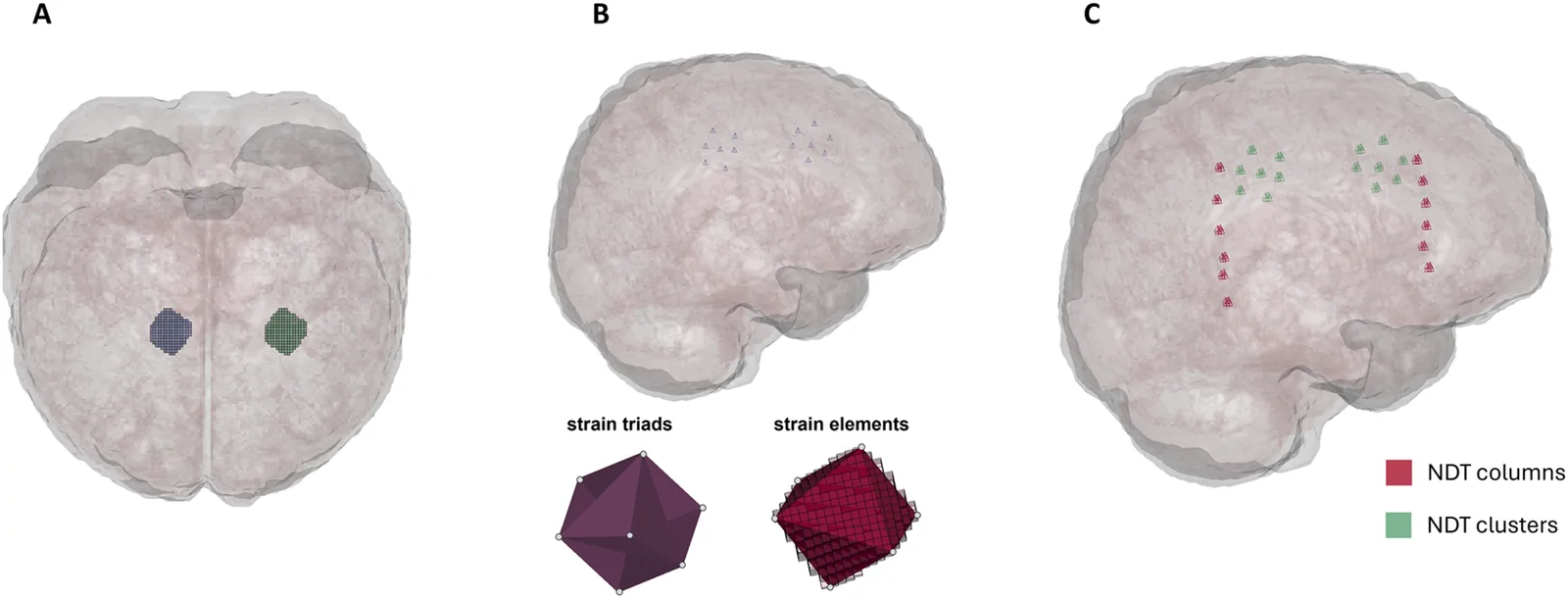
Selected elements used to calculate coup ICP response (blue elements) and contrecoup ICP response (green elements) for right temporal impact C393-T3 **(A)**, location of clusters used for strain calculation and visualization of strain triad elements generated from the cluster nodes in purple and selected brain element representing brain tissue encompassed by the cluster in red **(B)**, and NDT implantation showing column (C383-T1) and cluster (C288-T3) configurations **(C)**.

The goal of the current study is to develop and validate a realistic model of the brain-skull interface. This, along with a number of other improvements and refinements, have been implemented into the ABM, resulting in the release of a new version of the ABM - ABMv4. The ability of the ABMv4 to predict stresses, strains, and local displacements experienced by the brain in real-world head impact events is then compared to the performance of the original ABM and, when available, other existing FE models.

## 2 Materials and methods

The ABM originally developed by Miller et al. (2016) has been updated and modified in several ways including mesh and geometry refinement, the introduction of a sliding contact at the skull and falx interfaces, and improved material properties (Miller et al., 2016). A new version, referred to as ABMv4, was developed to simulate and better predict brain response to wide-ranging head impacts (ABMv3 refers to an internal version of the model with a different sliding contact implemented at the CSF-skull interface that was never published). Model details including number of elements and material properties are reported in Supplementary Table S1.

### 2.1 Development of ABMv4

The previous version of the ABM was used as a baseline in the current study and will be referred to as ABMv2. The geometry of the original model was directly generated from the International Consortium for Brain Mapping (ICBM) (Mazziotta et al., 2001) brain atlas using a voxel-based mesh generation approach. The ABMv2 consisted of a total of seven parts: cerebrum, cerebellum, CSF, ventricles, falx, tentorium, and skull.

#### 2.1.1 Mesh and geometry refinement

The original cerebrum and CSF parts were separated into right and left hemispheres and modified to be quasi-symmetric so that the hemispheres could be bisected by a planar falx. Furthermore, a layer of CSF elements was incorporated on each side of the falx to aid in the contact definition and ensure there were no locations where the falx was in direct contact with brain elements without any CSF between.

The center of gravity (CG) is established with reference to the Frankfurt plane, which is defined by two anatomic landmarks: (1) the external auditory meatus (EAM) and (2) the infraorbital margin (IOM). Because the location of the CG with reference to the EAM varies between individuals based on head size and shape, a literature review was conducted to determine the average location of the CG relative to the EAM and IOM. The CG was determined to be 10.18 mm anterior and 27.48 mm superior to the EAM (Becker, 1972; Walker et al., 1973; Beier et al., 1980; Albery and Whitestone, 2003). This represents a 2.64 mm change in CG location from the previous version of the model.

#### 2.1.2 Sliding contact

The interface at the skull of the ABM was improved by implementing a sliding contact between the skull and CSF parts. This was accomplished by generating a smooth skull and adding a layer of tetrahedral CSF elements. The smooth skull was developed in LS-PrePost v4.8.31 (ANSYS, Inc., Livermore, CA) using the ‘wrap’ method of element generation. Hypermesh (Altair Hyperworks, Troy, MI) was then used to generate a layer of tetrahedral CSF elements between the previous outer surface of the CSF (consisting of all cubic elements) and the new skull. Once the mesh was generated, element quality was assessed using element quality criteria based on Global Human Body Models Consortium (GHBMC) standards, with tet collapse given top priority (Elemance, 2021; Davis et al., 2015). The tet collapse criterion was set with the goal of 100% adherence; for all other element quality standards, a 99% adherence standard was set. These element quality guidelines were based on Global Human Body Models Consortium (GHBMC) standards (Elemance, 2021; Davis et al., 2015).

Sliding contact was then implemented at the new skull interface using *CONTACT_AUTOMATIC_SURFACE_TO_SURFACE_TIEBREAK. To apply proper interaction at this boundary, the tiebreak option in LS-DYNA was set to 4, which allows tangential sliding, but limits separation between the interfaces. Additionally, both the static coefficient of friction (FS) and dynamic coefficient of friction (FD) were set to 0.0, the soft constraint parameter (SOFT) was set to 1, and the scale factor for constrain forces of soft constrain option (SOFSCL) was set to 0.001. Default values were used for all other contact parameters.

The falx geometry was also updated and extended to join the new skull geometry and a sliding interface was also implemented between the falx and the adjacent CSF using the same method as at the skull.

### 2.2 Validation tests

A set of 23 experimental cadaver tests (Table 1) were used for model validation based on the recommendations of Giordano and Kleiven (2016) (Giordano and Kleiven, 2016). Sixteen of the validation cases reported intracranial pressure (ICP) data, nine report brain strain, and seventeen reported brain motion/relative brain displacement data. A detailed description of the post-mortem human subject (PMHS) experimental setup is available elsewhere (Hardy et al., 2001, 2007; Hardy, 2007; Miller et al., 2016). Three-dimensional (3D) kinematics of the head were measured experimentally and then used as boundary conditions for the simulations and applied about the CG of the ABMv4. Before simulation of the experimental tests, the ABM was scaled to match the head dimensions of the respective PMHSs for each test and simulations were run using LS-DYNA (ANSYS, Inc., Livermore, CA). Scale factors were determined for each subject from the average of the following two ratios: (1) subject head depth compared to ABM head depth and (2) subject head breadth compared to ABM head breadth. The model was then uniformly scaled in all directions using the calculated scale factor.

**TABLE 1:** Summary of the test configurations used for validation of the ABMv4.

​	Impact configuration			Lin acc	Rot acc	Rot vel	Output data		
Test	Plane	Location	NDTs	(g)	(rad/s^2^)	(rad/s)	ICP	Disp	Strain	References
C064-T4	Sagittal	Occipital	Clusters (2)	122	6,669	16.6	✓	✓	✓	Hardy et al., 2007
C241-T5	Sagittal	Occipital	Clusters (2)	194	15,261	15.9	✓	✓	✓	Hardy et al., 2007
C241-T6	Sagittal	Occipital	Clusters (2)	127	13,509	18.3	✓	✓	✓	Hardy et al., 2007
C288-T3	Sagittal	Occipital	Clusters (2)	236	33,491	30.2	✓	✓	✓	Hardy et al., 2007
C291-T1	Coronal	R parietal	Columns (3)	162	7,222	24.9	​	✓	​	Hardy 2007
C380-T4	Coronal	L temporal	Clusters (2)	196	18,655	37.8	✓	✓	✓	Hardy et al., 2007
C380-T5	Horizontal	L parietal	Clusters (2)	77	6,284	25.3	✓	✓	✓	Hardy et al., 2007
C380-T6	Coronal	L temporal	Clusters (2)	147	13,020	31.7	✓	✓	✓	Hardy et al., 2007
C383-T1	Sagittal	Frontal	Columns (2)	63	2,718	20.3	​	✓	​	Hardy et al., 2001
C383-T3	Sagittal	Frontal	Columns (2)	65	3,549	21.9	​	✓	​	Hardy et al., 2001
C383-T4	Sagittal	Occipital	Columns (2)	927	29,701	21.3	​	✓	​	Hardy et al., 2001
C393-T3	Coronal	L temporal	Clusters (2)	159	16,939	26.6	✓	✓	✓	Hardy et al., 2007
C393-T4	Coronal	L temporal	Clusters (2)	180	14,473	28.5	✓	✓	✓	Hardy et al., 2007
C428-T1	Sagittal	Frontal	Columns (2)	75	6,079	18.4	​	✓	​	Hardy 2007
C755-T2	Sagittal	Occipital	Columns (2)	22	1,744	17.8	​	✓	​	Hardy et al., 2001
C755-T5	Sagittal	Frontal	Columns (2)	11	876	7.1	​	✓	​	Hardy et al., 2001
C787-T1	Sagittal	Frontal	Columns (2)	54	2,991	16.6	✓	​	​	Hardy 2007
C787-T2	Sagittal	Frontal	Columns (2)	325	16,733	19.2	✓	​	​	Hardy 2007
C910-T1	Coronal	R parietal	Columns (3)	487	43,406	36.4	✓	✓	​	Hardy 2007
C910-T2	Horizontal	R occipital	Columns (3)	74	4,335	27.7	✓	​	​	Hardy 2007
C910-T3	Horizontal	R occipital	Columns (3)	79	4,069	28.3	✓	​	​	Hardy 2007
C916-T1	Horizontal	R occipital	Columns (3)	120	10,227	24.2	✓	​	​	Hardy 2007
C916-T2	Horizontal	R occipital	Columns (3)	75	6,648	21.8	✓	​	​	Hardy 2007

#### 2.2.1 Intracranial pressure (ICP) tests

In the intracranial pressure experiments, ICP was measured using two implanted cranial pressure transducers implanted in the brain tissue at sites approximating the coup and contrecoup locations (Hardy, 2007). For example, for the C393-T3 test, which is an impact to the left temporal lobe, the elements used to estimate coup and contrecoup ICP response are shown in Figure 1A.

ICP pressure, *p*, was calculated as the negative of hydrostatic stress, which is the average of the principal stresses and is defined as:

$$\sigma_{h}=\frac{\sigma_{1}+\sigma_{2}+\sigma_{3}}{3}$$

The principal stresses are calculated from the Cauchy stress tensor, which is directly output by LS-DYNA for each brain element at each time point, by identifying the principal directions where the corresponding stress vectors are normal to the planes (Livermore Software Technology Corporation, 2006).

#### 2.2.2 Strain tests

The brain strain was estimated using the 3D coordinate data from the NDT cluster targets. Hardy et al., 2007 used LS-DYNA to prescribe the motion of each NDT modeled as a single node and connected to neighboring nodes to form triads (3-node shell elements). The number of triads was variable in time depending on the availability of tracked data for the targets that formed a given triad. When the center target was available, up to twelve triads were formed within the cluster and when the center target data were missing, up to eight triads were formed (Figure 1B). Maximum principal strain and maximum shear strain were directly computed from the shell deformations at each time point from the nodal data and then triad strain was calculated using a custom MATLAB script; results from all available triads were averaged for each NDT cluster.

In addition to the original strain data published by Hardy, revised strain values from Zhou et al. (2019a) for a subset of tests are also examined and used for validation purposes (Table 2). Zhou et al. (2019b) recalculated brain strain by imposing the measured NDT motion to an NDT tetrahedral model (Zhou et al., 2019b). This reanalysis and recalculation of strain was performed using tetrahedral elements because it is known that experimental 3D strain is not well-represented by planar elements (Waldman et al., 1985). To compare to the revised strain data, brain elements encompassed by each NDT cluster (Figure 1B) were selected and the strain was determined directly from the LS-DYNA output (ELOUT), based on an approach previously described by Zhou et al., 2019b.

**TABLE 2:** Data availability summary for strain validation tests.

Test	Cluster 1		Cluster 2	
	Hardy et al. (2007)	Zhou et al. (2019b)	Hardy et al. (2007)	Zhou et al. (2019b)
C064-T4	0–30 ms	0–20 ms	0–65 ms	--
C241-T5	0–35 ms	--	0–50 ms	0–15 ms
C241-T6	0–60 ms	--	0–60 ms	0–40 ms
C288-T3	0–120 ms	0–105 ms	0–120 ms	0–40 ms
C380-T4	0–120 ms	0–120 ms	0–120 ms	--
C380-T5	0–15 ms	0–20 ms	0–15 ms	--
C380-T6	0–120 ms	0–120 ms	0–120 ms	--
C393-T3	0–60 ms	0–50 ms	0–95 ms	--
C393-T4	0–120 ms	0–35 ms	0–120 ms	--

#### 2.2.3 Brain-skull displacement tests

The brain-skull relative displacement tests were conducted using high-speed biplanar X-ray to track the relative motion of implanted radio-opaque neutral density targets (NDTs) implanted in either columns or cluster configurations (Hardy et al., 2001). The NDTs were implanted in a variety of configurations and locations depending on the test condition, but, in general, used either a column or cluster configuration. Of the seventeen considered in the current study, eight used NDTs implanted in 2–3 vertical columns and nine used a cluster configuration. Most cases using NDT columns were impacts in the sagittal plane (C383, C428, C755) and exhibited one anterior NDT column (AC) placed in the frontal lobe and one posterior column (PC) placed in the parietal lobe, both in the right hemisphere. The remaining two cases with NDT columns (C291 and C910) were both parietal impacts in the coronal plane and exhibited 3 NDT columns: one in the right and left parietal lobe, respectively, and one in the left frontal lobe for C291-T1 and one in the R frontal lobe for C910-T1. The remaining impacts used NDT cluster configuration, which consisted of 7 NDTs in each cluster. For the sagittal plane impacts (C064, C241, C288), the first NDT cluster (C1) was placed in the frontal lobe and the second (C2) in the parietal lobe, both in the right hemisphere. The C1 cluster was placed in the right parietofrontal lobe and the C2 cluster in the left parietofrontal lobe for the remaining impacts (C380 and C393). Figure 1C show an example of both NDT implantation schemes.

### 2.3 Model performance

To evaluate model performance, error between experimental and predicted experimental curves was quantified using CORA (CORrelation and Analysis) Version 3.6 (Gehre et al., 2009). Following the protocol of Giordano and Kleiven (2016), the current study will only use the results from the cross-correlation sub-method using the parameters listed in Supplementary Table S2 (Giordano and Kleiven, 2016). The sliding scale for the total rating point of CORA is defined in Technical Report ISO/TR 979038 as follows: ‘‘Excellent’’: 0.86–1.0, ‘‘Good’’: 0.65–0.86, ‘‘Fair’’: 0.44–0.65, ‘‘Marginal’’: 0.26–0.44, and ‘‘Unacceptable’’: 0.0–0.26 (ISO/TR, 1999).

### 2.4 Head impact simulations

Two youth football impacts and two collegiate soccer headers were simulated with both versions of the ABM and the strain responses compared. The impacts were selected to most closely approximate a 50th and 95th percentile impact based on full-season linear acceleration and rotational velocity data collected with instrumented mouthpieces. Additional details on data collection, instrumentation, and impact selection are included in the Supplementary Material. The strain distributions were analyzed for both ABMv2 and ABMv4 and a suite of strain metrics calculated based on previous work with the ABM (Miller et al., 2021, 2022). From this set of strain metrics, MPS_1,95_ will be used in the current study. MPS_1,95_ is calculated by determining the 95th percentile strain value from the distribution of strain values at each time point, resulting in a time history curve of 95th percentile strain values; the MPS_1,95_ metric is then taken as the maximum of that curve. Additionally, the difference in surface strains between the two versions of the model will be evaluated by calculating the strain (and relevant metrics) in just the surface brain elements, as shown in Figure 2. The surface elements were defined as any brain element within 4 mm of the closest skull element, which resulted in 77,564 elements (or 12.8% of the gray matter volume).

**FIGURE 2:**
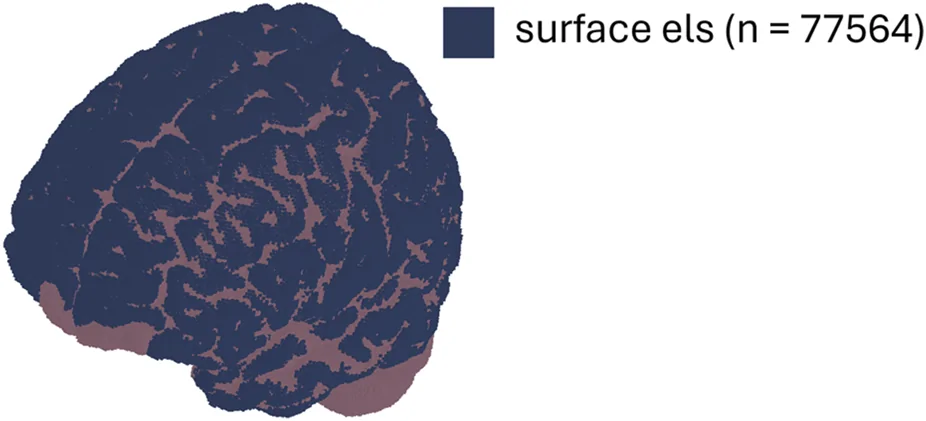
Brain surface elements used to evaluate the difference in surface strains between ABMv2 and ABMv4.

## 3 Results

### 3.1 Enhancement of ABM

A smooth skull was generated by ‘wrapping’ shell elements around the ABMv2 skull geometry in LS-PrePost. Using a target element length of 1 mm (to facilitate the transition between the new tetrahedral mesh and the original 1 × 1 × 1 cubic mesh), a new surface was generated consisting of 269,820 shell elements (with an enclosed volume of 1,601.5 mL). A tetrahedral mesh was then generated to fill the space between the new and old skull surfaces, resulting in 1,091,240 tetrahedral elements. The new part was defined as extra-axial CSF with a volume of 143.7 mL. Cross sections of the ABMv2 and ABMv4 brain-skull interfaces are shown in Figure 3.

**FIGURE 3:**
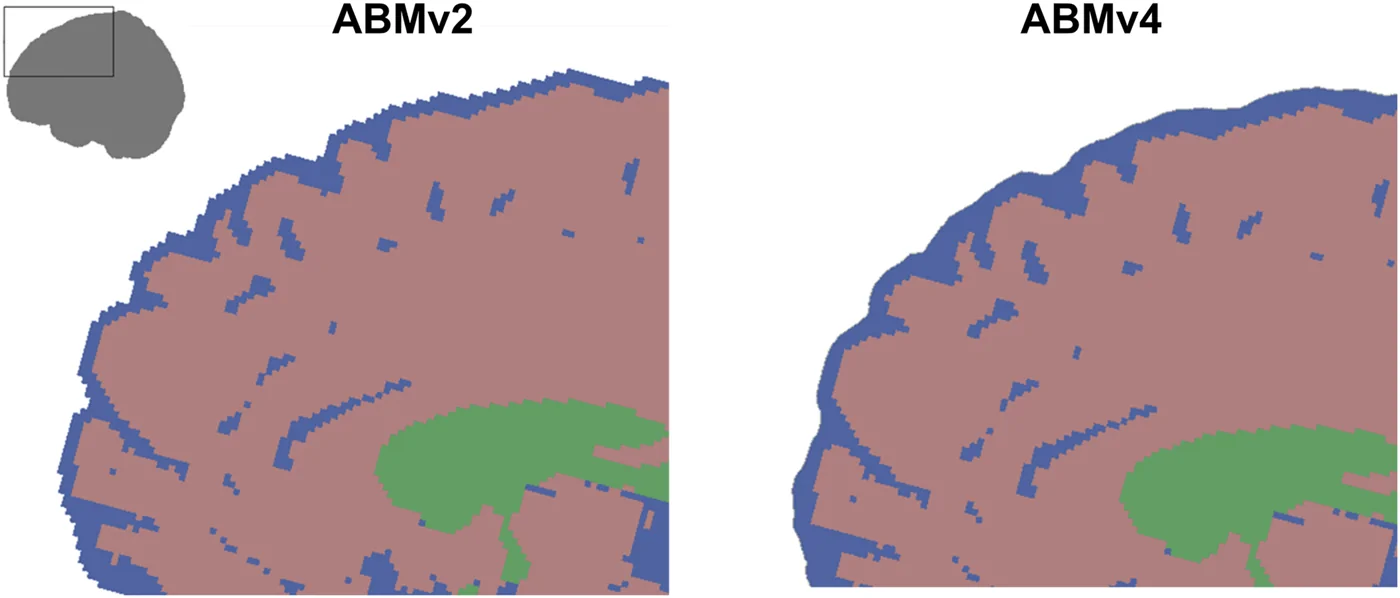
Zoomed-in cross sections of the old ABM and the new, decoupled ABM with a smooth skull surface.

### 3.2 Validation results

The ABMv4 was validated against 23 experimental cadaver impacts–sixteen ICP tests, nine strain tests, and seventeen brain displacement tests. The scaled models ranged in skull and extra-axial CSF volume from 1,766 to 2,524 mL and 158.5 to 226.5 mL, respectively. Coup and contrecoup ICP plots for all tests are reported in Supplementary Figures S1-S3 and the CORA scores in Table 3. Principal and shear strain results for all nine tests are reported in Supplementary Figures S4-S5 and the corresponding CORA scores in Table 4. CORA scores calculated using the revised strain curves from (Zhou et al., 2019b) are also reported in Table 4. Selected NDT displacement results are reported in Supplementary Figures S8-S24 and all average CORA results are reported in Table 5.

**TABLE 3:** ICP CORA results.

​	Test	Coup ICP	Contrecoup ICP	Ave
1	C064-T4	--	0.841	0.841
2	C241-T5	0.864	0.638	0.751
3	C241-T6	0.748	0.819	0.784
4	C288-T3	0.674	--	0.674
5	C380-T4	--	0.506	0.506
6	C380-T5	--	0.868	0.868
7	C380-T6	--	0.653	0.653
8	C393-T3	0.733	--	0.733
9	C393-T4	0.687	--	0.687
10	C787-T1	0.917	0.633	0.775
11	C787-T2	0.711	0.520	0.616
12	C910-T1	0.521	0.435	0.478
13	C910-T2	0.944	0.855	0.900
14	C910-T3	0.949	0.794	0.872
15	C916-T1	0.908	0.629	0.769
16	C916-T2	0.915	0.712	0.814
​	​	​	​	0.733

**TABLE 4:** Strain CORA results.

​	Test	Original strain				​	​	Revised strain	
		C1		C2		​	C1		C2		​
		Prin	Shear	Prin	Shear	Ave	Prin	Shear	Prin	Shear	Ave
1	C064-T4	0.564	0.566	0.498	0.531	0.540	0.728	0.685	--	--	0.707
2	C241-T5	0.586	0.589	0.655	0.666	0.624	--	--	0.841	0.803	0.822
3	C241-T6	0.403	0.629	0.331	0.399	0.441	--	--	0.857	0.805	0.831
4	C288-T3	0.573	0.703	0.361	0.327	0.491	0.983	0.901	0.795	0.841	0.880
5	C380-T4	0.737	0.763	0.710	0.729	0.735	0.827	0.928	--	--	0.878
6	C380-T5	0.582	0.595	0.586	0.593	0.589	0.907	0.860	--	--	0.884
7	C380-T6	0.506	0.701	0.690	0.707	0.651	0.774	0.854	--	--	0.814
8	C393-T3	0.684	0.695	0.341	0.37	0.523	0.980	0.972	--	--	0.976
9	C393-T4	0.443	0.466	0.471	0.495	0.469	0.804	0.842	--	--	0.823
​	​	​	​	​	​	0.563	​	​	​	​	0.846

**TABLE 5:** Relative brain displacement/NDT CORA results.

​	Test	X	Y	Z	Ave
1	C064-T4	0.492	0.519	0.595	0.521
2	C241-T5	0.761	0.600	0.649	0.670
3	C241-T6	0.676	0.600	0.454	0.577
4	C288-T3	0.716	0.652	0.620	0.663
5	C291-T1	--	0.704	0.571	0.637
6	C380-T4	0.709	0.561	0.649	0.639
7	C380-T5	0.535	0.463	0.507	0.502
8	C380-T6	0.670	0.516	0.666	0.617
9	C383-T1	0.557	--	0.588	0.573
10	C383-T3	0.487	--	0.608	0.548
11	C383-T4	0.567	--	0.674	0.620
12	C393-T3	0.710	0.855	0.573	0.713
13	C393-T4	0.806	0.807	0.748	0.787
14	C428-T1	0.537	--	0.488	0.513
15	C755-T2	0.772	--	0.597	0.684
16	C755-T5	0.671	--	0.370	0.520
17	C910-T1	--	0.484	0.510	0.497
​	​	​	​	​	0.605

Relative displacement was also quantified and analyzed along the brain-skull interface. Node pairs were defined between all nodes on the brain surface and the closest skull node. It should be noted that this measure does not distinguish tangential sliding from changes in the normal gap, but the change in normal distance is assumed to be negligible since the contact definition restricts separation between the outer surface of the CSF and the skull. Relative displacements on the surface of the brain are shown in Figure 4 for ABMv2 and ABMv4 for three of the validation tests. The shaded areas represent the full range of relative displacements from all approximately 45,000 brain-skull node pairs for the ABMv2 (blue) and ABMv4 (red) models. The relative displacement plots were generated by first identifying all nodes on the brain surface (n = 42,936) and then determining the closest skull node. Then relative displacement was calculated at each time point as the distance between the paired brain-skull nodes minus the initial distance.

**FIGURE 4:**
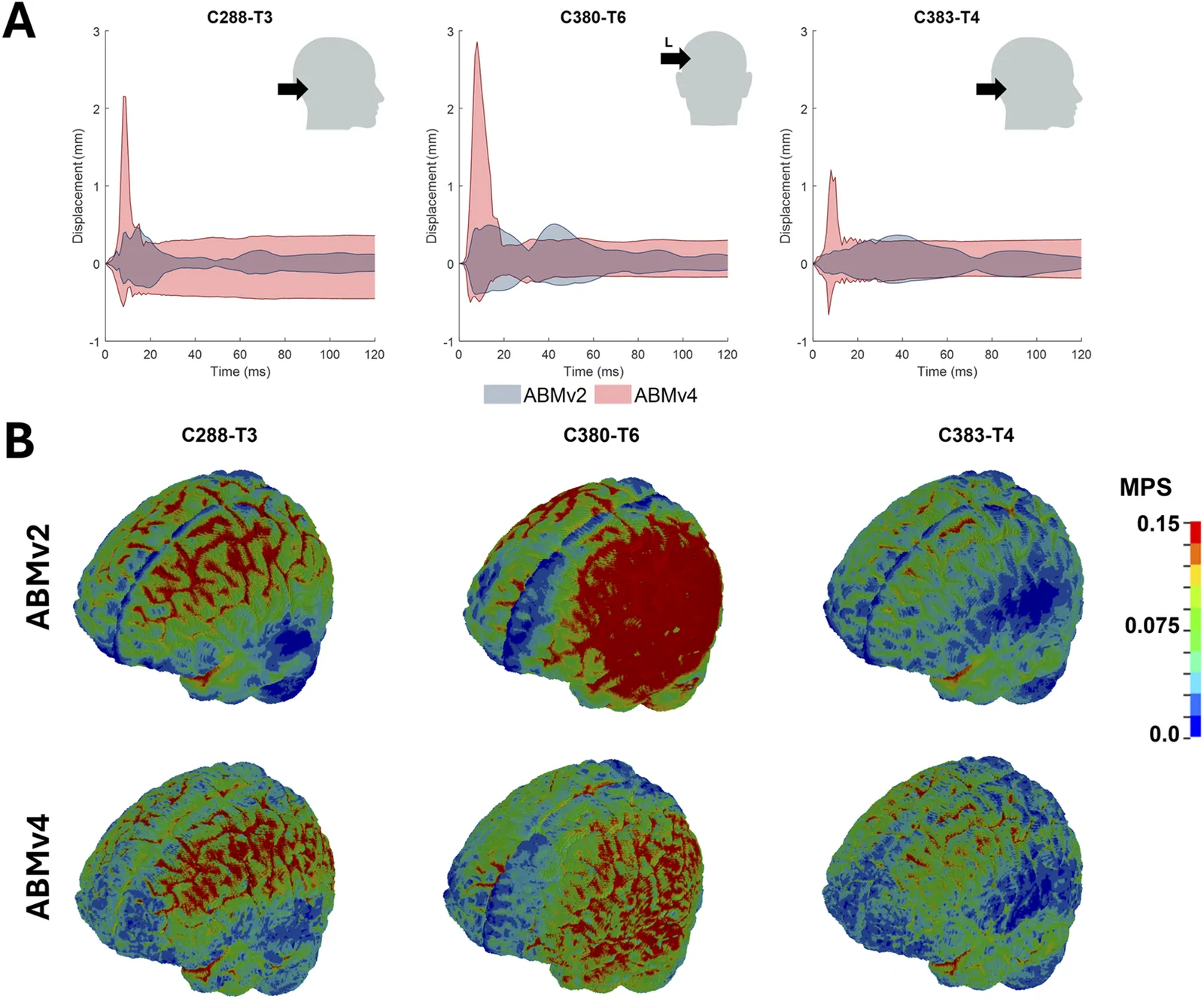
Comparison of ABMv2 and ABMv4 relative brain-skull displacement **(A)** and surface strain **(B)** in three of the hardy validation tests.

### 3.3 Results of head impact simulations

Four real-world head impacts were simulated with both the ABMv2 and ABMv4 for comparison purposes. The peak kinematics for the peak kinematics for the 50th/95th youth football impacts were 20.9/45.7 g, 9.0/16.2 rad/s, and 1,589/5,547 rad/s^2^, respectively; the 50th/95th percentile collegiate soccer headers were 8.3/25.8 g, 4.0/9.5 rad/s, and 418/2,428 rad/s^2^, respectively. The strain contours at the time of peak strain are shown for both models for the 50th% football impact in Figure 5A and for the 95th% soccer impact in Figure 5B.

**FIGURE 5:**
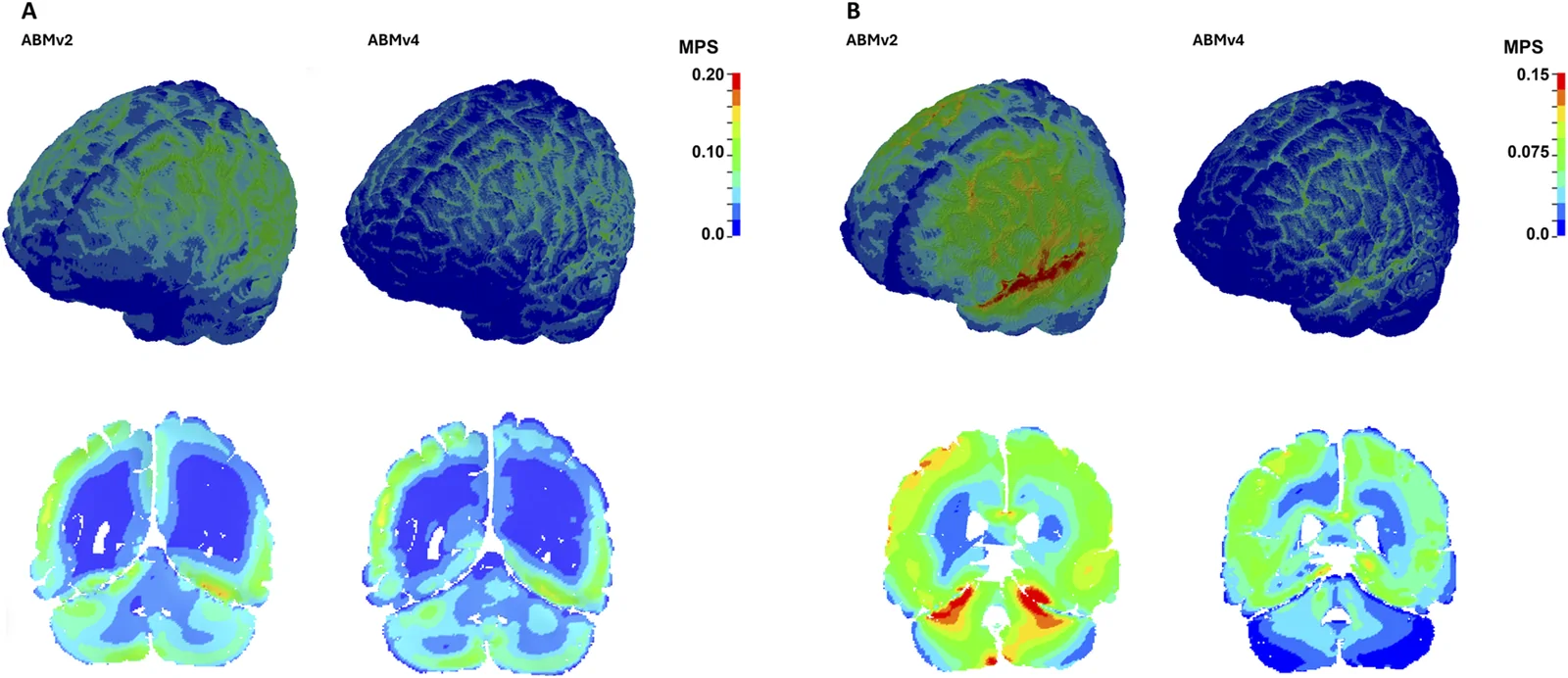
Strain contours at time of maximum strain (t = 30 ms) for ABMv2 (left) and ABMv4 (right) for the 50th% youth football head contact event **(A)** and for the 95th% collegiate soccer header **(B)**.

## 4 Discussion

The brain-skull interface of the ABM was improved in the current study by decoupling the skull and CSF, implementing a smooth skull geometry, and defining a sliding contact between the skull and CSF. Figure 4A shows the difference in magnitude of relative displacement along the brain-skull interface in the old ABM compared to the decoupled model (ABMv4). The maximum relative displacement in the decoupled ABM is approximately five times greater than the maximum displacement in ABMv2 for the three shown validation tests. Additionally, Figure 4B shows that changes to ABMv4 increased the relative motion at the brain-skull interface, with a concurrent reduction in surface strains. While there were additional changes made from ABMv2 to ABMv4 (such as modified falx and cerebral hemispheric geometry and updated head CG location) that may be partially responsible for the performance improvement as well as the increase in relative displacement along the brain-skull interface, some of difference in responses between, given the magnitude of the changes associated with the newly skull-CSF interface, the current study has attributed the largest changes to the sliding contact. The effort to decouple the brain and skull was undertaken to improve the biofidelity of this important interface since it is often modeled, as in the ABMv2, by representing the CSF as solid with a very low shear modulus (Zhou et al., 1995, 2016; Ho et al., 2009; Mao et al., 2013; Iwamoto and Nakahira, 2015; Zhao et al., 2015). This formulation allows some relative movement between the brain and skull through deformation of the low-shear CSF elements, but can cause excessive element distortion, which can result in low accuracy and small time step sizes (Aquelet et al., 2006). Zhou et al. (2019c) used a fluid-structure interaction (FSI) approach in an updated version of the original KTH head model, referred to as KTH-FSI model, which modifies CSF elements in the subarachnoid space using arbitrary Lagrangian-Eulerian (ALE) multi-material formulation (Zhou et al., 2019a). This approach achieved improved validation performance when compared to the original KTH model, which uses a sliding interface (Kleiven, 2007). Additionally, when subjected to a subset of the Hardy PMHS experiments, the KTH-FSI model showed increased relative motion at the brain-skull interface (8.44 mm compared to 4.07 mm); it also showed, however, increased surface strains (0.008 compared to 0.004) (Zhou et al., 2019a). The FSI approach does, however, come with increased computation time - Zhou et al., 2019a reported the FSI model took 9 h to run a 40 ms impact on a single processor, while the sliding model took 5 h, representing an 80% increase in computation time. The increased computational cost, along with the increased resolution and anatomic detail in the ABM (the ABM has approximately ∼150 times the number of nodes and elements as the KTH-FSI model) result in FSI not being a practical approach for the ABM (Giudice et al., 2019).

The ABMv4 was validated against 23 cadaver impact experiments. The validation of the model using three distinct kinds of experimental data suggests that the ABMv4 is accurate at predicting the overall response kinematics of the head during a range of impact velocities and locations. To our knowledge, this is the first time a brain model has been validated against the complete suite of experimental tests recommended by (Giordano and Kleiven, 2016). The overall biofidelity rating of the ABMv4 for the cases tests ranged from 0.441 to 0.900 with an average value of 0.644 (when considering the strain validation against the original Hardy strain data) and is comparable to similar values previously reported (Giordano and Kleiven, 2016; Trotta et al., 2020). The ICP CORA scores ranged from 0.478 to 0.900 with an average value of 0.733 (evaluated as “Good”) and the NDT displacement CORA scores ranged from 0.497 to 0.787 with an average value of 0.605 (evaluated as “Fair”). When considering the original Hardy strain data, the CORA scores ranged from 0.441 to 0.735 with an average value of 0.563 (evaluated as “Fair”), but when comparing against the revised strain data the CORA scores ranged from 0.707 to 0.976 with an average value of 0.846 (evaluated as “Good”). Figure 6 shows the difference in magnitude between the originally reported Hardy strain values and those revised by (Zhou et al., 2019b) for the C288-T3 experiment. For the nine experiments where strain data was analyzed, the revised principal strain and shear strains were on average 6.0 and 5.6 times greater than their original values due to employing a tetrahedral estimation approach as opposed to the triad-based approach used by Hardy et al. (2007). The fidelity of tetra-based approach was evaluated using three independently validation brain FE models and observed good agreement between the tetra-calculated strain and measured FE strain values (Zhou et al., 2019b). Additionally, the tetra-estimated strain values exhibit good agreement with results from physical models, supporting the use of this approach for the validation of brain FE models intended for strain prediction (Margulies et al., 1990; Meaney et al., 1995; Zou et al., 2007). We believe the revised strain values are a truer representation of brain deformation and strain, as found by Zhou et al. (2019b), but we have included comparisons to both to allow easier comparison to the performance of other models, since the original Hardy strain is much more commonly used in validation studies (Giordano and Kleiven, 2016; Zhou et al., 2019c).

**FIGURE 6:**
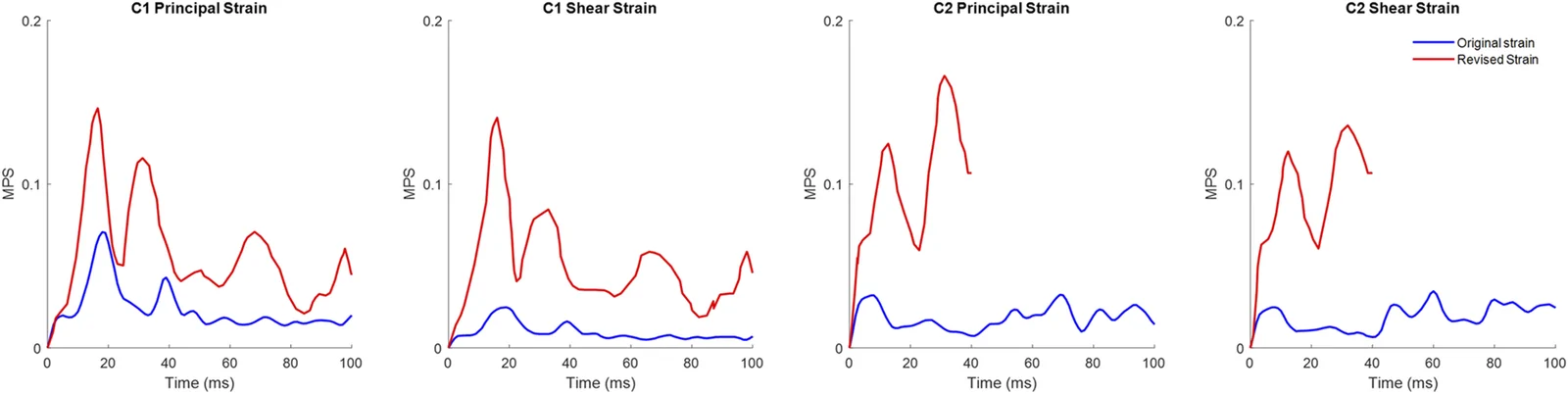
Comparison of original and revised strain curves for C288-T3.

Average CORA scores for each type of validation test (ICP, strain, NDT displacement) are summarized in Table 6 and compared to previously published CORA scores for four selected models: two versions of the KTH model (Kleiven, 2007; Giordano and Kleiven, 2014; Giordano et al., 2014), the Total Human Model for Safety (THUMS) head model version 4.02 (Kimpara et al., 2006), and the Global Human Body Model Consortium (GHBMC) head model version 4.4 (Mao et al., 2013; Giordano and Kleiven, 2016). CORA scores for selected NDT displacement tests are also included for the ABMv2 version of the model from previous validation work (Miller et al., 2017). From this table, we see that when considering only ICP tests, the THUMS model performs the best; when considering only strain tests, the KTH models perform the best; when considering only the NDT displacement tests, the ABM performs the best; and when considering all three kinds of tests overall, the KTH anisotropic model performs the best.

**TABLE 6:** CORA results for all validation cases compared to performance of other FEMs.

​	Test	KTH ISO	KTH ANISO	THUMS	GHBMC	ABMv2	ABMv4
ICP tests (n = 16)	C064-T4	0.761	0.797	0.826	0.827	0.741	0.841
	C241-T5	0.855	0.751	0.714	0.630	0.638	0.753
	C241-T6	0.605	0.836	0.628	0.531	0.621	0.784
	C288-T3	0.761	0.797	0.826	0.827	0.529	0.674
	C380-T4	0.340	0.349	0.492	0.653	0.408	0.506
	C380-T5	0.699	0.531	0.672	0.666	0.231	0.868
	C380-T6	0.589	0.661	0.691	0.704	0.656	0.653
	C393-T3	0.707	0.781	0.834	0.819	0.670	0.733
	C393-T4	0.890	0.901	0.815	0.719	0.606	0.688
	C787-T1	--	--	--	--	0.639	0.775
	C787-T2	--	--	--	--	0.785	0.616
	C910-T1	--	--	--	--	0.651	0.478
	C910-T2	--	--	--	--	0.504	0.900
	C910-T3	--	--	--	--	0.521	0.872
	C916-T1	--	--	--	--	0.455	0.769
	C916-T2	--	--	--	--	0.426	0.814
​	ICP average	0.690	0.712	0.722	0.708	0.568	0.733
Strain tests (n = 9)	C064-T4	0.704	0.713	0.428	0.350	0.494	0.540
	C241-T5	0.681	0.782	0.578	0.642	0.619	0.624
	C241-T6	0.829	0.838	0.615	0.469	0.472	0.441
	C288-T3	0.766	0.643	0.499	0.612	0.440	0.491
	C380-T4	0.635	0.624	0.704	0.419	0.715	0.735
	C380-T5	0.560	0.518	0.355	0.486	0.585	0.589
	C380-T6	0.731	0.767	0.609	0.544	0.689	0.651
	C393-T3	0.565	0.550	0.557	0.432	0.499	0.523
	C393-T4	0.637	0.675	0.521	0.455	0.581	0.469
​	Strain average	0.679	0.679	0.541	0.490	0.566	0.563
Revised strain tests (n = 9)	C064-T4	--	--	--	--	0.731	0.707
	C241-T5	--	--	--	--	0.779	0.822
	C241-T6	--	--	--	--	0.292	0.831
	C288-T3	0.595	--	--	--	0.621	0.880
	C380-T4	0.568	--	--	--	0.872	0.878
	C380-T5	--	--	--	--	0.871	0.884
	C380-T6	0.522	--	--	--	0.871	0.814
	C393-T3	--	--	--	--	0.888	0.976
	C393-T4	--	--	--	--	0.913	0.823
Strain (revised) average		0.562	--	--	--	0.760	0.846
NDT displacement tests (n = 17)	C064-T4	0.376	0.533	0.456	0.467	0.547	0.521
	C241-T5	0.529	0.691	0.491	0.541	0.693	0.670
	C241-T6	0.344	0.447	0.603	0.552	0.607	0.577
	C288-T3	0.498	0.615	0.590	0.634	0.654	0.663
	C291-T1	--	--	--	--	0.572	0.637
	C380-T4	0.525	0.490	0.540	0.627	0.715	0.639
	C380-T5	0.617	0.576	0.551	0.512	0.516	0.502
	C380-T6	0.564	0.563	0.537	0.615	0.730	0.617
	C383-T1	--	--	--	--	0.624	0.573
	C383-T3	--	--	--	--	0.628	0.548
	C383-T4	--	--	--	--	0.613	0.620
	C393-T3	0.668	0.670	0.610	0.661	0.689	0.713
	C393-T4	0.586	0.571	0.641	0.660	0.800	0.787
	C428-T1	--	--	--	--	0.446	0.513
	C755-T2	--	--	--	--	0.662	0.684
	C755-T5	--	--	--	--	0.492	0.520
	C910-T1	--	--	--	--	0.506	0.497
​	NDT average	0.523	0.573	0.558	0.585	0.617	0.605
Overall average (original MPS)		0.630	0.654	0.607	0.595	0.587	0.644
Overall average (revised MPS)		0.600	0.642	0.640	0.647	0.629	0.705

The first MPS section in Table 6 uses the original Hardy strain data to calculate the reported CORA values, while the second MPS section takes into account the updated strain measures from Zhou et al. (2019a). When considering the CORA scores calculated from the updated strain values, the ABM outperforms all other models in both the strain only tests, with an average strain CORA score of 0.846, and the overall average, with an overall average CORA score of 0.705. We do not have access to the complete performance and CORA scores of the KTH, THUMS, and GHBMC models against the reanalyzed strain data except for a few exceptions which were digitized from Zhou et al. (2019a) and used to calculate KTH CORA scores relative to the updated strain data (Figure 7) (Zhou et al., 2019c). The average principal and shear strain CORA scores for the three available tests, C288-T3, C380-T4, C380-T6, for the ABMv4 were 0.880, 0.878, and 0.814, respectively, and for the KTH model were 0.595, 0.568, and 0.522, respectively. This gives as average performance of 0.857 for the ABMv4 and 0.562 for the KTH when considering the revised strain data.

**FIGURE 7:**
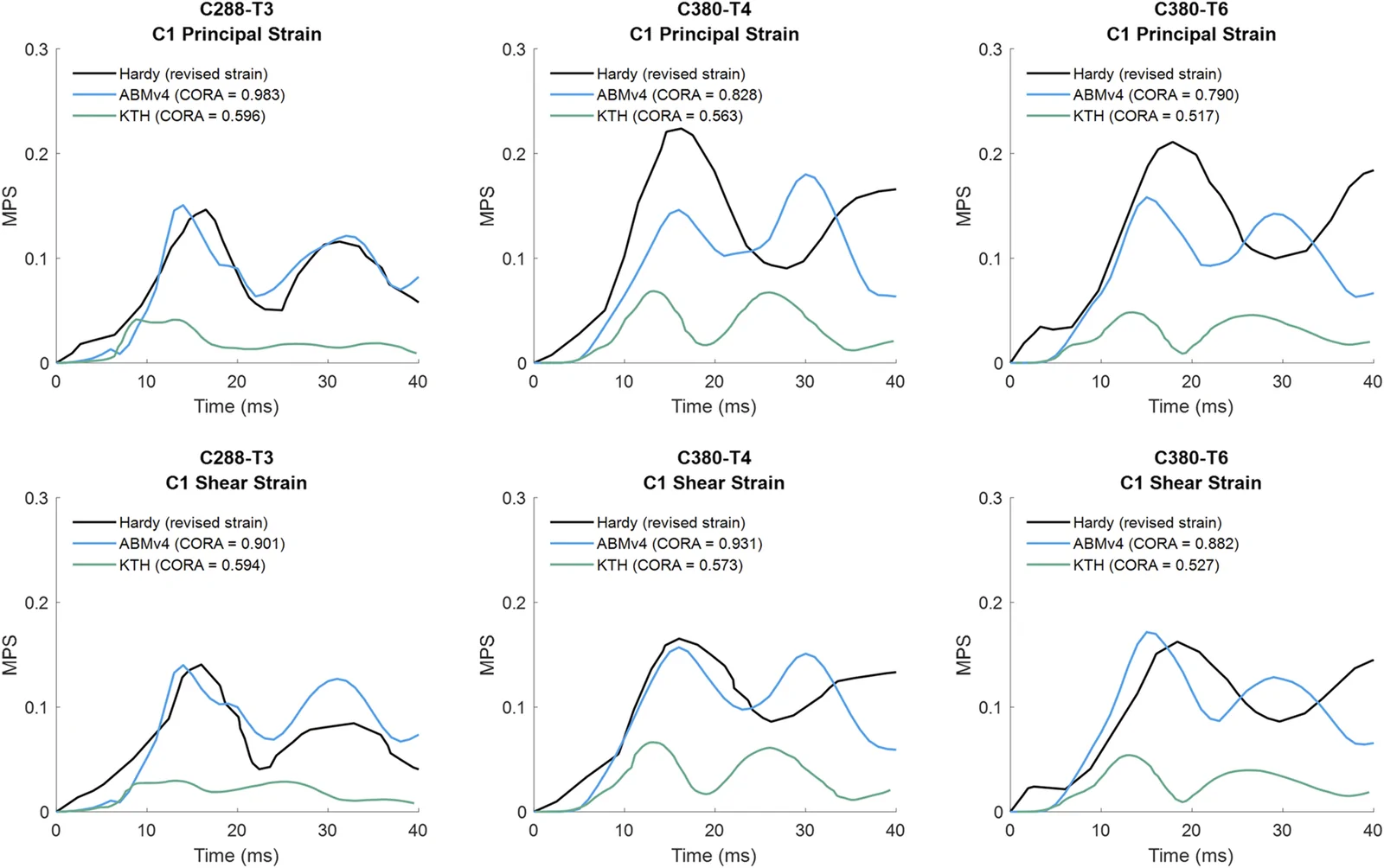
Strain response compared to the revised strain data for the ABMv4 and KTH models for C288-T3, C380-T4, and C380-T6.

Comparing the strain responses to the real-world head impact events, it is evident that the pattern of surface strain distribution is consistent between ABMv2 and ABMv4, but the strain magnitude, particularly on the brain surface, is reduced for ABMv4. This is explained by the newly incorporated sliding contact between the brain and skull. This sliding interface allows the CSF elements to move tangentially relative to the skull surface, rather than distorting and transferring that load to the elements on the surface of the brain. The overall strain is slightly increased in ABMv4 compared to ABMv2 (average MPS_1,95_ value of 0.1489 for ABMv4 compared to 0.1409for ABMv2); the surface strains, however, show a more decrease, with an average MPS_1,95_ value of 0.0921 for ABMv4 compared to 0.1462 for ABMv2 (Table 7). While the reduction in surface brain strain in ABMv4 is in harmony with expected biofidelic behavior, this finding alone does not definitely prove an improved biofidelic response compared with the results of ABMv2 as there are no high-rate experimental measurements of brain surface strain to compare against, which is a limitation of the current work.

**TABLE 7:** MPS_1,95_ values for ABM model versions.

​	Full brain		Surface	
	ABMv2	ABMv4	ABMv2	ABMv4
50th% youth football impact	0.0814	0.0711	0.0866	0.0476
95th% youth football impact	0.2322	0.2740	0.2406	0.1470
50th% collegiate soccer header	0.0791	0.0741	0.0812	0.0482
95th% collegiate soccer header	0.1707	0.1764	0.1762	0.1257

Despite improvements to the brain–skull interface, the ABMv4 still relies on several simplifying assumptions, including the use of an isotropic brain material model and homogeneous properties across the cerebrum and cerebellum. Additionally, there is some uncertainty about the CSF material properties and the best contact parameters for use in brain modeling. Limitations to the validation approach include subject variability and the absence of direct validation data on brain surface strain.

Future development of the ABM will include the incorporation of directional anisotropy, regional differences, and age dependence. Automatic fiber-tract quantification (AFQ) has been performed on the ICBM brain atlas to identify a total of 100 white matter (WM) fibers in each of 28 different WM tract groups (Wakana et al., 2004; Mori et al., 2008; Oishi et al., 2008; Yeatman et al., 2012). The WM fibers will be modeled with a total of 285,152 beam elements (ranging from 5,585 to 18,001 beam elements per tract group). The fibers will be modeled with a separate material model representing the mechanical properties of WM fiber bundles to accurately capture anisotropic response. Once appropriate material parameters have been identified for the WM fibers, the anisotropic ABM will then be re-validated.

### 4.1 Conclusion

The current study details model enhancements made to the ABM, including the addition of sliding contacts at the brain-skull and falx-CSF interfaces, mesh refinement, and enhancement of the CSF material properties. The brain-skull interface was enhanced by decoupling the current skull and CSF in the ABMv2 and implementing a smooth skull geometry for ABMv4. Additionally, contact was defined between the outer surface of the CSF and the skull. This improved model was validated against various ICP, brain strain, and brain displacement data and assessed using an objective rating method and achieved CORA ratings of 0.733, 0.563, 0.846, and 0.605 for ICP, brain strain, revised brain strain, and brain displacement validation tests, respectively. Additionally, the new version, ABMv4, results in increased relative brain-skull motion (average of 0.54 mm for ABMv2 compared to average of 1.06 mm for ABMv4) and reduced brain strain on the surface of the model, with the surface strain being on average 61.7% of the surface strain value predicted for the ABMv2. The new model performs well in all types of validation tests and achieves a good biofidelity rating.

## Data Availability

The raw data supporting the conclusions of this article will be made available by the authors, without undue reservation.

## References

[B1] Albery C. Whitestone J. (2003). Comparison of cadaveric human head mass properties: mechanical measurement vs. calculation from medical imaging., 157–172.

[B2] Alshareef A. Wu T. Giudice J. S. Panzer M. B. (2021). Toward subject-specific evaluation: methods of evaluating finite element brain models using experimental high-rate rotational brain motion. Biomech. Model. Mechanobiol. 20, 2301–2317. 10.1007/s10237-021-01508-7 34432184

[B3] Aquelet N. Souli M. Olovsson L. (2006). Euler–Lagrange coupling with damping effects: application to slamming problems. Comput. Methods Appl. Mech. Eng. 195, 110–132. 10.1016/j.cma.2005.01.010

[B4] Baeck K. (2013). Biomechanical Modeling of Head Impacts – A Critical Analysis of Finite Element Modeling Approaches. Belgium: KU Leuven.

[B5] Barnes-Wood M. McCloskey H. Connelly S. Gilchrist M. Annaidh A. N. Theobald P. (2024). Investigation of head kinematics and brain strain response during soccer heading using a custom-fit instrumented mouthguard. Ann. Biomed. Eng. 52, 934–945. 10.1007/s10439-023-03430-8 38243139 PMC10940496

[B6] Bayly P. V. Cohen T. S. Leister E. P. Ajo D. Leuthardt E. C. Genin G. M. (2005). Deformation of the human brain induced by mild acceleration. J. Neurotrauma 22, 845–856. 10.1089/neu.2005.22.845 16083352 PMC2377024

[B7] Becker E. B. (1972). Measurement of mass distribution parameters of anatomical segments. SAE Trans. 81, 2818–2833. 10.4271/720964

[B8] Beier G. Schuller E. Schuck M. Ewing C. Becker E. Thomas D. (1980). Center of gravity and moments of inertia of human heads.

[B9] Belingardi G. Chiandussi G. Gaviglio I. (2005). Development and validation of a new finite element model of human head.

[B10] Chafi M. S. Dirisala V. Karami G. Ziejewski M. (2009). A finite element method parametric study of the dynamic response of the human brain with different cerebrospinal fluid constitutive properties. Proc. Inst. Mech. Eng. H. 223, 1003–1019. 10.1243/09544119JEIM631 20092097

[B11] Chen Y. Sutton B. Conway C. Broglio S. P. Ostoja-Starzewski M. (2012). Brain deformation under mild impact: magnetic resonance imaging-based assessment and finite element study.

[B12] Davis M. L. Koya B. Stitzel J. D. Gayzik F. S. (2015). Development of the GHBMC 5th percentile female finite element model. 10.4271/2016-22-0015 27871105

[B13] Dixit P. Liu G. (2017). A review on recent development of finite element models for head injury simulations. Arch. Comput. Methods Eng. 24, 979–1031. 10.1007/s11831-016-9196-x

[B14] Elkin B. S. Gabler L. F. Panzer M. B. Siegmund G. P. (2019). Brain tissue strains vary with head impact location: a possible explanation for increased concussion risk in struck *versus* striking football players. Clin. Biomech. 64, 49–57. 10.1016/j.clinbiomech.2018.03.021 29625747

[B500] Elkin B. S. Gabler L. F. Panzer M. B. Siegmund G. P. (2019). Brain tissue strains vary with head impact location: a possible explanation for increased concussion risk in struck *versus* striking football players. Clin. Biomech. 64, 49–57. 10.1016/j.clinbiomech.2018.03.021 29625747

[B84] Elemance LLC (2021). Global Human Body Models Consortium user manual: M50 detailed occupant version 6.0 for LS‐ DYNA. Elemance LLC.

[B15] Feng Y. Abney T. M. Okamoto R. J. Pless R. B. Genin G. M. Bayly P. V. (2010). Relative brain displacement and deformation during constrained mild frontal head impact. J. R. Soc. Interface 7, 1677–1688. 10.1098/rsif.2010.0210 20504801 PMC2988269

[B16] Fernandes F. A. Tchepel D. Alves de Sousa R. J. Ptak M. (2018). Development and validation of a new finite element human head model: yet another head model (YEAHM). Eng. Comput. 35, 477–496. 10.1108/ec-09-2016-0321

[B17] Filben T. M. Pritchard N. S. Miller L. E. Miles C. M. Urban J. E. Stitzel J. D. (2021). Header biomechanics in youth and collegiate female soccer. J. Biomech. 128, 110782. 10.1016/j.jbiomech.2021.110782 34656012

[B18] Gehre C. Gades H. Wernicke P. (2009). Objective rating of signals using test and simulation responses., 09–0407.

[B19] Ghajari M. Hellyer P. J. Sharp D. J. (2017). Computational modelling of traumatic brain injury predicts the location of chronic traumatic encephalopathy pathology. Brain 140, 333–343. 10.1093/brain/aww317 28043957 PMC5278309

[B20] Giordano C. Kleiven S. (2014). Evaluation of axonal strain as a predictor for mild traumatic brain injuries using finite element modeling. Stapp Car Crash J. 58, 29–61. 10.4271/2014-22-0002 26192949

[B21] Giordano C. Kleiven S. (2016). Development of an unbiased validation protocol to assess the biofidelity of finite element head models used in prediction of traumatic brain injury. 10.4271/2016-22-0013 27871103

[B22] Giordano C. Cloots R. J. H. van Dommelen J. A. W. Kleiven S. (2014). The influence of anisotropy on brain injury prediction. J. Biomech. 47, 1052–1059. 10.1016/j.jbiomech.2013.12.036 24462379

[B23] Giudice J. S. Zeng W. Wu T. Alshareef A. Shedd D. F. Panzer M. B. (2019). An analytical review of the numerical methods used for finite element modeling of traumatic brain injury. Ann. Biomed. Eng. 47, 1855–1872. 10.1007/s10439-018-02161-5 30377899

[B24] Giudice J. S. Alshareef A. Wu T. Gancayco C. A. Reynier K. A. Tustison N. J. (2020). An image registration-based morphing technique for generating subject-specific brain finite element models. Ann. Biomed. Eng. 48, 2412–2424. 10.1007/s10439-020-02584-z 32725547

[B25] Giudice J. S. Alshareef A. Wu T. Knutsen A. K. Hiscox L. V. Johnson C. L. (2021). Calibration of a heterogeneous brain model using a subject-specific inverse finite element approach. Front. Bioeng. Biotechnol. 9, 664268. 10.3389/fbioe.2021.664268 34017826 PMC8129184

[B26] Gomes M. S. Carmo G. P. Ptak M. Fernandes F. A. Alves de Sousa R. J. (2024). Accuracy and efficiency of finite element head models: the role of finite element formulation and material laws. Int. J. Numer. Methods Biomed. Eng. 40, e3851. 10.1002/cnm.3851 39045773

[B27] Hardy W. N. (2007). Response of the Human Cadaver Head to Impact. Wayne State University.

[B28] Hardy W. N. Foster C. D. Mason M. J. Yang K. H. King A. I. Tashman S. (2001). Investigation of head injury mechanisms using neutral density technology and high-speed biplanar X-ray. Stapp Car Crash J. 45, 337–368. 10.4271/2001-22-0016 17458753

[B29] Hardy W. N. Mason M. J. Foster C. D. Shah C. S. Kopacz J. M. Yang K. H. (2007). A study of the response of the human cadaver head to impact. Stapp Car Crash J. 51, 17–80. 18278591 10.4271/2007-22-0002 PMC2474809

[B30] Ho J. von Holst H. Kleiven S. (2009). Automatic generation and validation of patient-specific finite element head models suitable for crashworthiness analysis. Int. J. Crashworthiness 14, 555–563. 10.1080/13588260902895708

[B31] Holcomb J. M. Fisicaro R. A. Miller L. E. Yu F. F. Davenport E. M. Xi Y. (2021). Regional white matter diffusion changes associated with the cumulative tensile strain and strain rate in nonconcussed youth football players. J. Neurotrauma 38, 2763–2771. 10.1089/neu.2020.7580 34039024 PMC8820832

[B32] Horgan T. J. Gilchrist M. D. (2003). The creation of three-dimensional finite element models for simulating head impact biomechanics. IJCrash 8, 353–366. 10.1533/ijcr.2003.0243

[B33] ISO/TR (1999). Road Vehicles — Anthropomorphic Side Impact Dummy — Lateral Impact Response Requirements to Assess the Biofidelity of the Dummy.

[B34] Iwamoto M. Nakahira Y. (2015). Development and validation of the total HUman model for safety (THUMS) version 5 containing multiple 1D muscles for estimating occupant motions with muscle activation during side impacts. Stapp Car Crash J. 59, 53–90. 10.4271/2015-22-0003 26660740

[B35] Ji S. Zhu Q. Dougherty L. Margulies S. S. (2004). *In vivo* measurements of human brain displacement. Stapp Car Crash J. 48, 227–237. 10.4271/2004-22-0010 17230268

[B36] Ji S. Ghadyani H. Bolander R. P. Beckwith J. G. Ford J. C. McAllister T. W. (2014). Parametric comparisons of intracranial mechanical responses from three validated finite element models of the human head. Ann. Biomed. Eng. 42, 11–24. 10.1007/s10439-013-0907-2 24077860 PMC4397967

[B37] Ji S. Ghajari M. Mao H. Kraft R. H. Hajiaghamemar M. Panzer M. B. (2022). Use of brain biomechanical models for monitoring impact exposure in contact sports. Ann. Biomed. Eng. 50, 1389–1408. 10.1007/s10439-022-02999-w 35867314 PMC9652195

[B38] Jin X. Ma C. Zhang L. Yang K. H. King A. I. Dong G. (2007). Biomechanical response of the bovine pia-arachnoid complex to normal traction loading at varying strain rates. Stapp Car Crash J. 51, 115–126. 10.4271/2007-22-0004 18278593

[B39] Karton C. Blaine Hoshizaki T. Gilchrist M. D. (2020). A novel repetitive head impact exposure measurement tool differentiates player position in National Football League. Sci. Rep. 10, 1200. 10.1038/s41598-019-54874-9 31992719 PMC6987098

[B40] Khasawneh A. H. Garling R. J. Harris C. A. (2018). Cerebrospinal fluid circulation: what do we know and how do we know it? Brain Circ. 4, 14–18. 10.4103/bc.bc_3_18 30276331 PMC6057699

[B41] Kimpara H. Nakahira Y. Iwamoto M. Miki K. Ichihara K. Kawano S. (2006). Investigation of anteroposterior head-neck responses during severe frontal impacts using a brain-spinal cord complex FE model. Stapp Car Crash J. 50, 509–544. 10.4271/2006-22-0019 17311175

[B42] Kleiven S. (2007). Predictors for traumatic brain injuries evaluated through accident reconstructions. 1, 10.4271/2007-22-0003 18278592

[B43] Kleiven S. Hardy W. N. (2002). Correlation of an FE model of the human head with local brain motion-consequences for injury prediction. Stapp Car Crash J. 46, 123–144. 10.4271/2002-22-0007 17096222

[B44] Levy Y. Bian K. Patterson L. Ouckama R. Mao H. (2021). Head kinematics and injury metrics for laboratory hockey-relevant head impact experiments. Ann. Biomed. Eng. 49, 2914–2923. 10.1007/s10439-021-02855-3 34472000

[B45] Li Z. Yang H. Wang G. Han X. Zhang S. (2019). Compressive properties and constitutive modeling of different regions of 8-week-old pediatric porcine brain under large strain and wide strain rates. J. Mech. Behav. Biomed. Mater. 89, 122–131. 10.1016/j.jmbbm.2018.09.010 30268868

[B46] Li X. Zhou Z. Kleiven S. (2021). An anatomically detailed and personalizable head injury model: significance of brain and white matter tract morphological variability on strain. Biomech. Model. Mechanobiol. 20, 403–431. 10.1007/s10237-020-01391-8 33037509 PMC7979680

[B47] Livermore Software Technology Corporation (LSTC) (2006). LS-DYNA theory manual.

[B48] Lyu D. Zhou R. Lin C. Prasad P. Zhang L. (2022). Development and validation of a new anisotropic visco-hyperelastic human head finite element model capable of predicting multiple brain injuries. Front. Bioeng. Biotechnol. 10, 831595. 10.3389/fbioe.2022.831595 35402400 PMC8987584

[B49] Mao H. Zhang L. Jiang B. Genthikatti V. V. Jin X. Zhu F. (2013). Development of a finite element human head model partially validated with thirty five experimental cases. J. Biomech. Eng. 135, 111002. 10.1115/1.4025101 24065136

[B50] Margulies S. S. Thibault L. E. Gennarelli T. A. (1990). Physical model simulations of brain injury in the primate. J. Biomech. 23, 823–836. 10.1016/0021-9290(90)90029-3 2384494

[B51] Mazziotta J. Toga A. Evans A. Fox P. Lancaster J. Zilles K. (2001). A probabilistic atlas and reference system for the human brain: International Consortium for Brain Mapping (ICBM). Philos. Trans. R. Soc. Lond. B. Biol. Sci. 356, 1293–1322. 10.1098/rstb.2001.0915 11545704 PMC1088516

[B52] Meaney D. F. Smith D. H. Shreiber D. I. Bain A. C. Miller R. T. Ross D. T. (1995). Biomechanical analysis of experimental diffuse axonal injury. J. Neurotrauma 12, 689–694. 10.1089/neu.1995.12.689 8683620

[B53] Milef G. M. Miller L. E. DiGuglielmo D. M. Payne T. D. Filben T. M. Urban J. E. (2022). Head impact kinematics and brain deformation in paired opposing youth football players. J. Appl. Biomech. 38, 136–147. 10.1123/jab.2021-0098 35483702

[B54] Miller L. E. Urban J. E. Stitzel J. D. (2016). Development and validation of an atlas-based finite element brain model. Biomech. Model. Mechanobiol. 15, 1201–1214. 10.1007/s10237-015-0754-1 26762217 PMC4942406

[B55] Miller L. E. Urban J. E. Stitzel J. D. (2017). Validation performance comparison for finite element models of the human brain. Comput. Methods Biomech. Biomed. Engin. 20, 1273–1288. 10.1080/10255842.2017.1340462 28701050 PMC5975353

[B56] Miller L. E. Urban J. E. Kelley M. E. Powers A. K. Whitlow C. T. Maldjian J. A. (2019). Evaluation of brain response during head impact in youth athletes using an anatomically accurate Finite element model. J. Neurotrauma 36, 1561–1570. 10.1089/neu.2018.6037 30489208 PMC6531907

[B57] Miller L. E. Urban J. E. Davenport E. M. Powers A. K. Whitlow C. T. Maldjian J. A. (2021). Brain strain: computational model-based metrics for head impact exposure and injury correlation. Ann. Biomed. Eng. 49, 1083–1096. 10.1007/s10439-020-02685-9 33258089 PMC10032321

[B58] Miller L. E. Urban J. E. Espeland M. A. Walkup M. P. Holcomb J. M. Davenport E. M. (2022). Cumulative strain-based metrics for predicting subconcussive head impact exposure–related imaging changes in a cohort of American youth football players. J. Neurosurg. Pediatr. 29, 387–396. 10.3171/2021.10.PEDS21355 35061991 PMC9404368

[B59] Mori S. Oishi K. Jiang H. Jiang L. Li X. Akhter K. (2008). Stereotaxic white matter atlas based on diffusion tensor imaging in an ICBM template. Neuroimage 40, 570–582. 10.1016/j.neuroimage.2007.12.035 18255316 PMC2478641

[B60] Nahum A. M. Smith R. Ward C. C. (1977). “Intracranial pressure dynamics during head impact,” in 21st Stapp Car Crash Conference (21st Stapp Car Crash Conference). Warrendale, PA: SAE International.

[B61] Oishi K. Zilles K. Amunts K. Faria A. Jiang H. Li X. (2008). Human brain white matter atlas: identification and assignment of common anatomical structures in superficial white matter. Neuroimage 43, 447–457. 10.1016/j.neuroimage.2008.07.009 18692144 PMC2586008

[B62] Raouf I. Sohail M. Kim H. S. Chung J.-H. (2025). A brief review of traumatic brain injury based on finite element modeling. Multiscale Sci. Eng. 7, 1–10. 10.1007/s42493-025-00131-3

[B64] Takhounts E. G. Eppinger R. H. Campbell J. Q. Tannous R. E. Power E. D. Shook L. S. (2003). On the development of the simon finite element head model. Stapp Car Crash J. 62, 293–318. 10.4271/2003-22-0007 17096247

[B65] Trosseille X. Tarriere C. Lavaste F. Guillon F. Domont A. (1992). Development of a FEM of the human head according to a specific test protocol. SAE Tech. Pap. 10.4271/922527

[B66] Trotta A. Clark J. M. McGoldrick A. Gilchrist M. D. Annaidh A. N. (2020). Biofidelic finite element modelling of brain trauma: importance of the scalp in simulating head impact. Int. J. Mech. Sci. 173, 105448. 10.1016/j.ijmecsci.2020.105448

[B67] Tse K. M. Lim S. P. Tan V. B. C. Lee H. P. (2014). A review of head injury and finite element head models. Am. J. Eng. Technol. Soc. 1, 28–52.

[B68] Wakana S. Jiang H. Nagae-Poetscher L. M. van Zijl P. C. M. Mori S. (2004). Fiber tract–based Atlas of Human white matter anatomy. Radiology 230, 77–87. 10.1148/radiol.2301021640 14645885

[B69] Waldman L. K. Funk Y. Covell J. W. (1985). Transmural myocardial deformation in the canine left ventricle. Normal *in vivo* three-dimensional finite strains. Circ. Res. 57, 152–163. 10.1161/01.res.57.1.152 4006099

[B70] Walker L. Harris E. Pontius U. (1973). “Mass, volume, center of mass, and mass moment of inertia of head and head and neck of human body,” in 16th Stapp Car Crash Conference (JSTOR). Warrendale, PA: SAE International.

[B71] Yang K. H. King A. I. (2011). “Modeling of the brain for injury simulation and prevention,” in Biomechanics of the Brain. Editor Miller K. (New York, NY: Springer), 91–110. 10.1007/978-1-4419-9997-9_5

[B72] Yeatman J. D. Dougherty R. F. Myall N. J. Wandell B. A. Feldman H. M. (2012). Tract profiles of white matter properties: automating fiber-tract quantification. PLoS ONE 7, e49790. 10.1371/journal.pone.0049790 23166771 PMC3498174

[B73] Zhang L. Yang K. H. Dwarampudi R. Omori K. Li T. Chang K. (2001). Recent advances in brain injury research: a new human head model development and validation. 10.4271/2001-22-0017 17458754

[B74] Zhao W. Ji S. (2019). Mesh convergence behavior and the effect of element integration of a human head injury model. Ann. Biomed. Eng. 47, 475–486. 10.1007/s10439-018-02159-z 30377900 PMC6344257

[B75] Zhao W. Ji S. (2020). Displacement-and strain-based discrimination of head injury models across a wide range of blunt conditions. Ann. Biomed. Eng. 48, 1661–1677. 10.1007/s10439-020-02496-y 32240424 PMC7286792

[B76] Zhao W. Ruan S. Ji S. (2015). Brain pressure responses in translational head impact: a dimensional analysis and a further computational study. Biomech. Model. Mechanobiol. 14, 753–766. 10.1007/s10237-014-0634-0 25412925 PMC4440857

[B77] Zhao W. Choate B. Ji S. (2018). Material properties of the brain in injury-relevant conditions – experiments and computational modeling. J. Mech. Behav. Biomed. Mater. 80, 222–234. 10.1016/j.jmbbm.2018.02.005 29453025 PMC5841256

[B78] Zhou C. Khalil T. B. King A. I. (1995). A new model comparing impact responses of the homogeneous and inhomogeneous human brain. Stapp Car Crash J. 1, 952714. 10.4271/952714

[B79] Zhou Z. Jiang B. Cao L. Zhu F. Mao H. Yang K. H. (2016). Numerical simulations of the 10-year-old head response in drop impacts and compression tests. Comput. Methods Programs Biomed. 131, 13–25. 10.1016/j.cmpb.2016.04.013 27265045

[B80] Zhou Z. Li X. Kleiven S. (2019a). Fluid–structure interaction simulation of the brain–skull interface for acute subdural haematoma prediction. Biomech. Model. Mechanobiol. 18, 155–173. 10.1007/s10237-018-1074-z 30151812 PMC6373285

[B81] Zhou Z. Li X. Kleiven S. Hardy W. N. (2019b). Brain strain from motion of sparse markers. Stapp Car Crash J. 63, 1–27. 10.4271/2019-22-0001 32311050

[B82] Zhou Z. Li X. Kleiven S. Shah C. S. Hardy W. N. (2019c). “A reanalysis of experimental brain strain data: implication for finite element head model validation,” in 62nd Stapp Car Crash Conference (Warrendale, PA: SAE International). 10.4271/2018-22-0007 30608998

[B83] Zou H. Schmiedeler J. P. Hardy W. N. (2007). Separating brain motion into rigid body displacement and deformation under low-severity impacts. J. Biomech. 40, 1183–1191. 10.1016/j.jbiomech.2006.06.018 16919640

