# Supplementary material for: Biomechanically informed improvements to the brain–skull interface in an atlas-based brain model
Source: Front Bioeng Biotechnol. 2026 Oct 29;14:1881544. doi: 10.3389/fbioe.2026.1881544 (PMC13757723; doi:10.3389/fbioe.2026.1881544)
Supplement: Supplementary file 1 [file Supplementaryfile1.pdf]

Supplementary Material

ABM Model Development

The original ABM FE model (Miller et al. 2016) has a volume of 1,639 mL, which is somewhat larger than the average reported by (Allen et al. 2002). This enlarged volume is expected because the ICBM average brain templates are slightly larger than average due to differences in brain shape and size (Toga and Mazziotta 2002). To better approximate the average brain, the model was uniformly scaled by a factor of 0.9195, by comparing the desired brain volume and the original volume using volume scaling (1,274/1,6393=0.9195). This results in a volume of 1,256 mL for the new scaled model. For validation tests, the ABM was scaled to match the subject anthropometry for each condition. The global scaling method, presented by Mertz et al. 1989, was used to calculate the scale factor for each condition, as it has previously been determined to be important for model validation (Horgan and Gilchrist 2003; Ji et al. 2015). Model details including number of elements, material models, and material parameters are reported in **Error! Reference source not found.**.

Table S1. Material Properties

Part	# elements	Material Model	Material Properties
Brain	1,635,864	Kelvin-Maxwell Viscoelastic	ρ = 1,040 kg/m^3<br>K = 2.19 GPa<br>G_0 = 4.06 kPa<br>G_∞ = 0.45 kPa
Ventricles	32,528	Elastic Fluid	ρ = 1,000 kg/m^3<br>K = 2.1 GPa<br>E = 0.0 kPa<br>ν = 0.5
Internal CSF	206,617	Elastic Fluid	ρ = 1,000 kg/m^3<br>K = 2.1 GPa<br>E = 0.0 MPa<br>ν = 0.5
Extra-Axial CSF	1,091,240	Kelvin-Maxwell Viscoelastic	ρ = 1,090 kg/m^3<br>K = 0.2 GPa<br>G_0 = 0.5 kPa<br>G_∞ = 0.1 kPa
Falx	10,562	Elastic	ρ = 1,130 kg/m^3<br>E = 157.9 MPa<br>ν = 0.45
Tentorium	13,082	Elastic	ρ = 1,130 kg/m^3<br>E = 31.5 MPa<br>ν = 0.45
Skull	269,820	Rigid	ρ = 22,310 kg/m^3<br>E = 11.5 MPa<br>ν = 0.3

Model Performance Evaluation

CORA (CORrelation and Analysis) Version 3.6 was used for quantitative evaluation of validation results in the current study (Gehre et al. 2009). The CORA parameters optimized for brain model validation by Giordano and Kleiven 2016 were used (**Error! Reference source not found.**) as opposed to the default CORA settings.

Table S2. CORA parameters

Parameter	Default	Pressure	Strain	Displacement
A_THRES	0.030	0.030	0.030	0.030
B_THRES	0.075	0.075	0.075	0.075
A_EVAL	0.010	0.010	0.010	0.010
B_DELTA_END	0.200	0.200	0.200	0.200
T_MIN, T_MAX	auto, auto	0, 30 ms	0, 40 ms	0, 40 ms
D_MIN	0.01	0.01	0.01	0.01
D_MAX	0.12	0.12	0.25	0.40
INT_MIN	0.80	0.70	0.70	0.80
K_V	10	3	3	3
K_P	1	1	1	1
K_G	1	1	1	1
G_V	0.50	0.33	0.33	0.33
G_P	0.25	0.33	0.33	0.33
G_G	0.25	0.33	0.33	0.33
G_1	0.5	0.0	0.0	0.0
G_2	0.5	1.0	1.0	1.0

Head Impact Data for Head Impact Simulations

Head impact data from two different studies conducted and approved by the Wake Forest School of Medicine Institutional Review Board (IRB) were sampled for use in the current study. The first study involved female soccer players from a National Collegiate Athletic Association Division I team and the second involved youth football athletes participating on two middle school level (ages 11-13) teams (Filben et al. 2022; Marks et al. 2022). All players were instrumented with a mouthpiece sensor containing a triaxial accelerometer and gyroscope embedded in acrylic, as described by Rich et al. 2019. From each dataset, 50th and 95th percentile linear acceleration and rotational velocity values were calculated and impacts most closely representing those values were selected for simulation. The 50th/95th percentile linear acceleration and rotational velocity values for the collegiate soccer data were 22.5/44.2 g and 9.1/16.2 rad/s, respectively; the 50th/95th percentile linear acceleration and rotational velocity values for the youth football data were 13.2/50.8 g and 10.1/22.4 rad/s, respectively. The peak kinematics for the selected impacts are shown in Table S3.

Table S3. Peak kinematics for selected head impacts for simulation

	Lin Acc (g)	Rot Vel (rad/s)	Rot Acc (rad/s^2)
50th percentile collegiate soccer header	20.9	9.0	1,589
95th percentile collegiate soccer header	45.7	16.2	5,547
50th percentile youth football impact	12.7	10.1	1,768
95th percentile youth football impact	48.8	22.4	7,549

Validation Results

Coup and contrecoup intracranial pressure (ICP) results are reported in **Error! Reference source not found.Error! Reference source not found.**. Principal and shear strain results employing the triad strain approach reported by Hardy et al. 2007 are reported in **Error! Reference source not found.** and **Error! Reference source not found.** while revised strain results from Zhou et al. 2019 are reported in **Error! Reference source not found.** and **Error! Reference source not found.**. Finally,

NDT displacement results are reported in **Error! Reference source not found.-Error! Reference source not found.**.

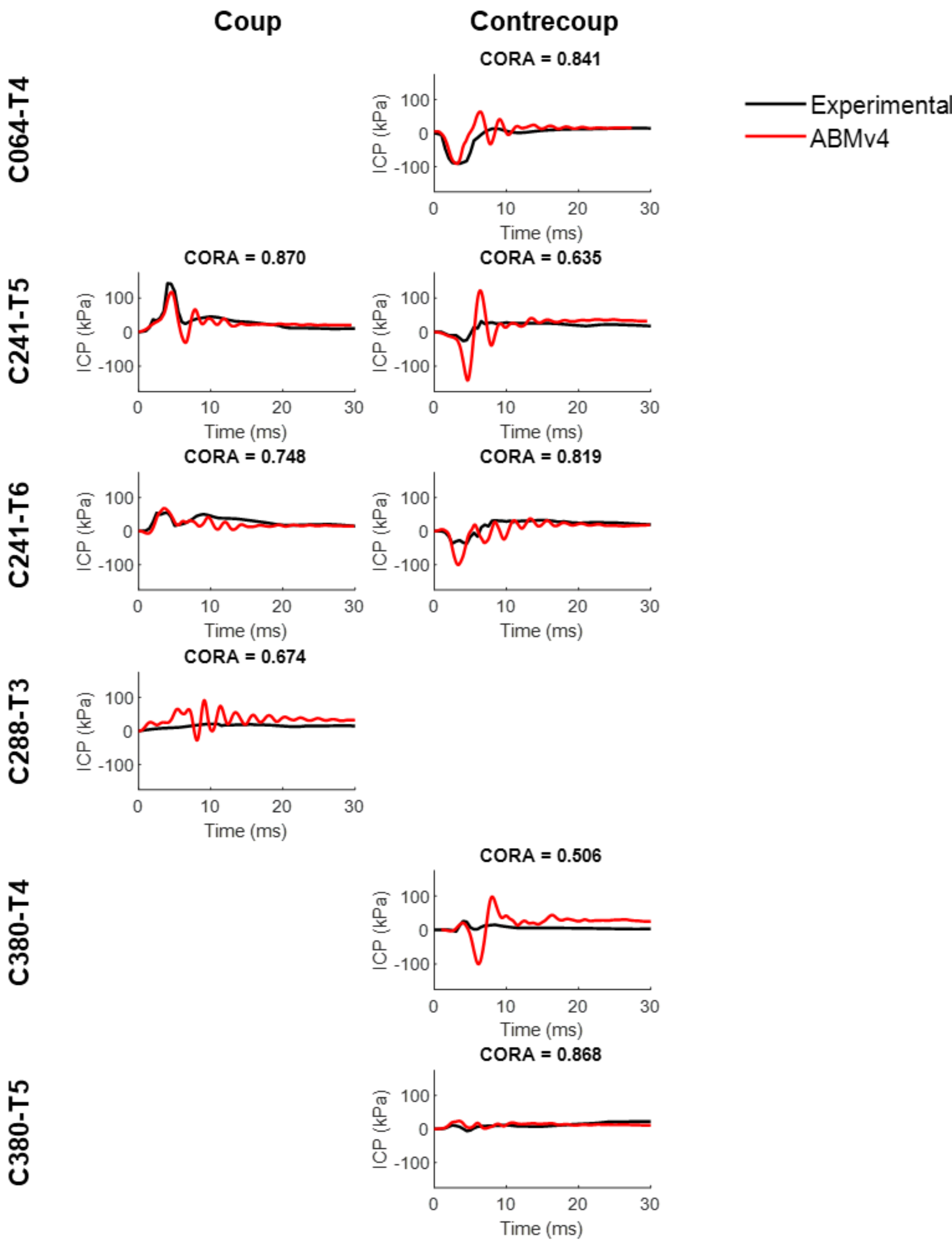

Figure S1. Comparison between experimental and ABMv4 simulated ICP responses at coup and contrecoup locations for experiments: C064-T4, C241-T5, C241-T6, C288-T3, C380-T4, and C380-T5.

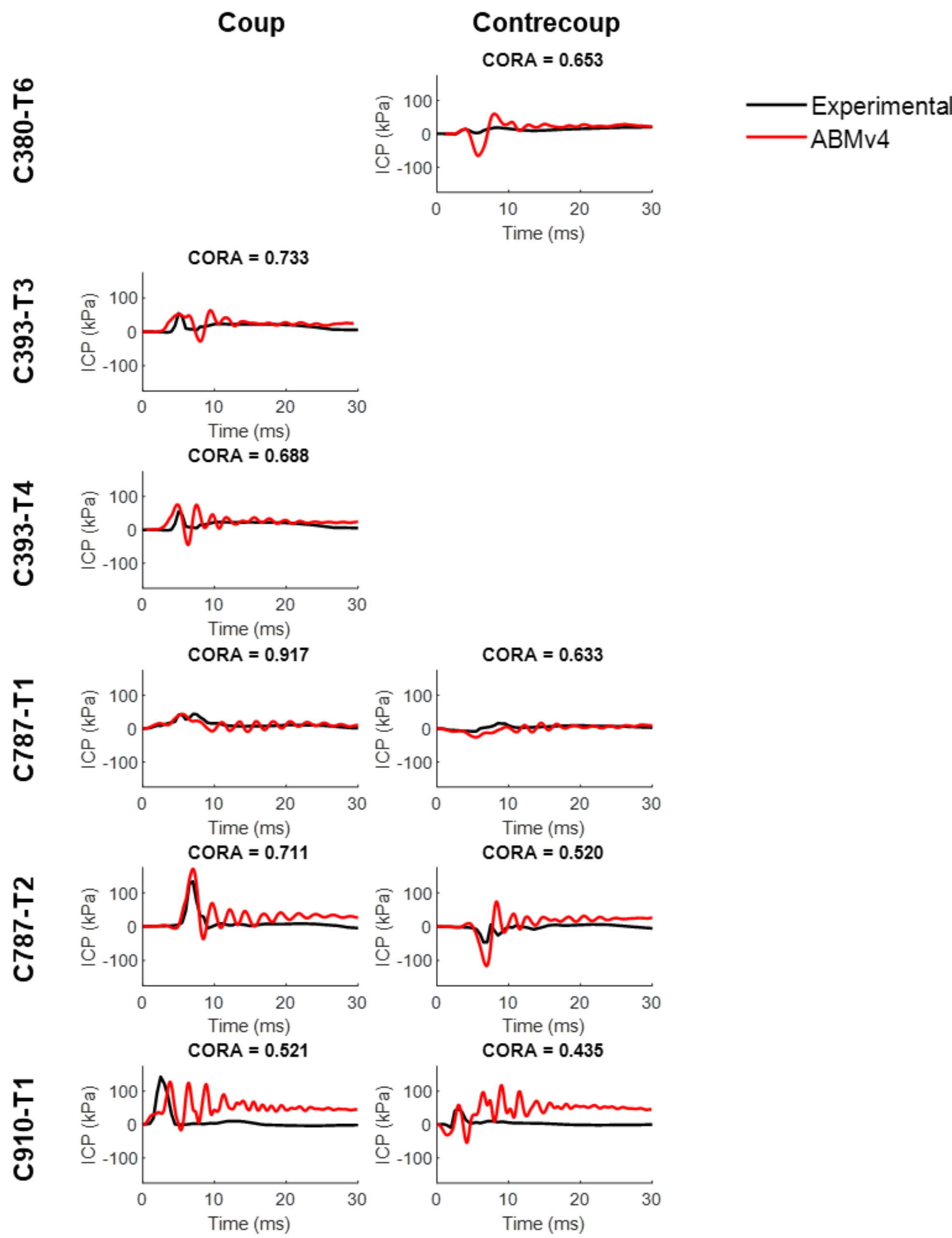

Figure S2. Comparison between experimental and ABMv4 simulated ICP responses at coup and contrecoup locations for experiments: C380-T6, C393-T3, C393-T4, C787-T1, C787-T2, and C910-T1.

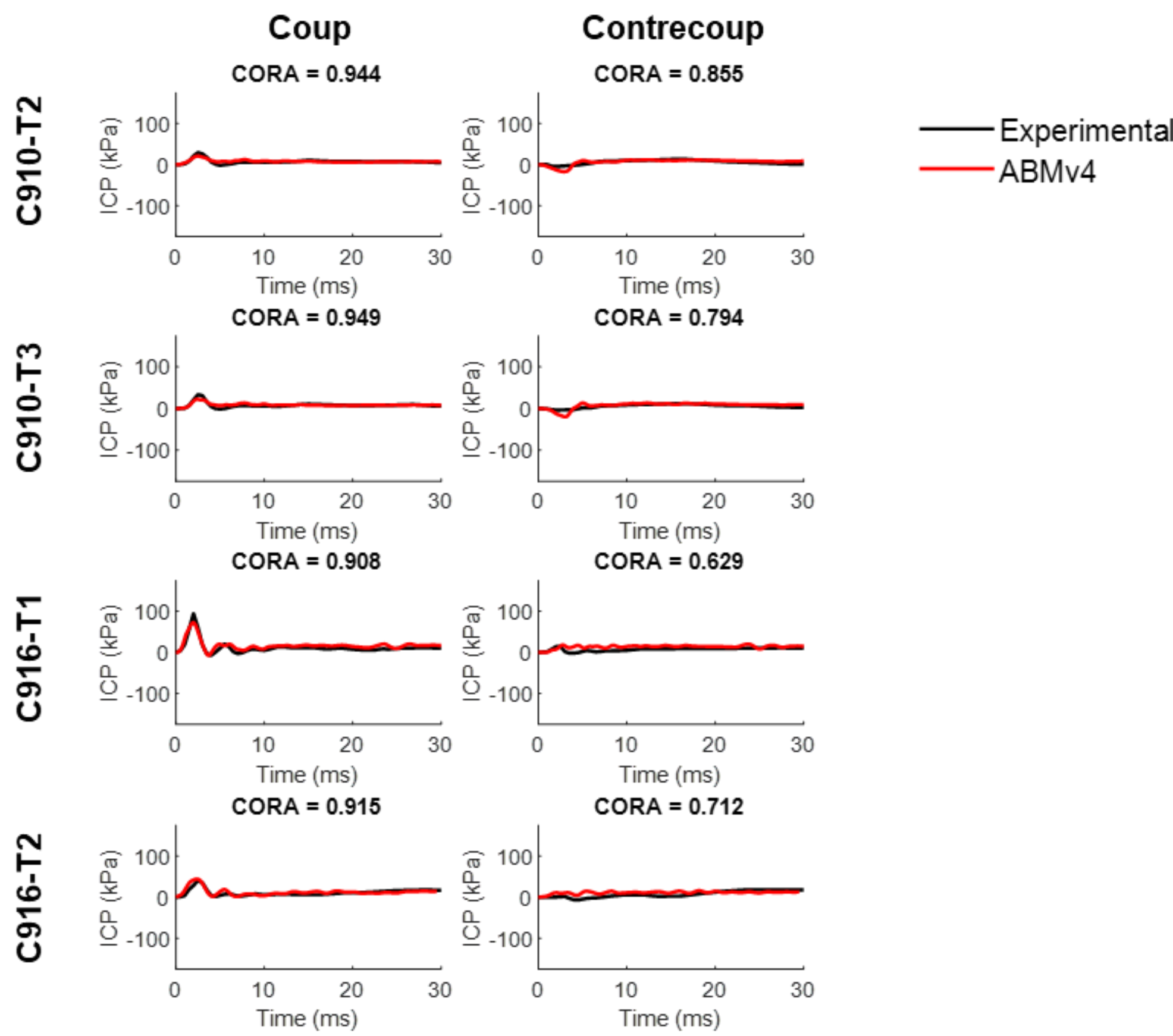

Figure S3. Comparison between experimental and ABMv4 simulated ICP responses at coup and contrecoup locations for experiments: C910-T1, C910-T2, C910-T3, C916-T1 and C916-T2.

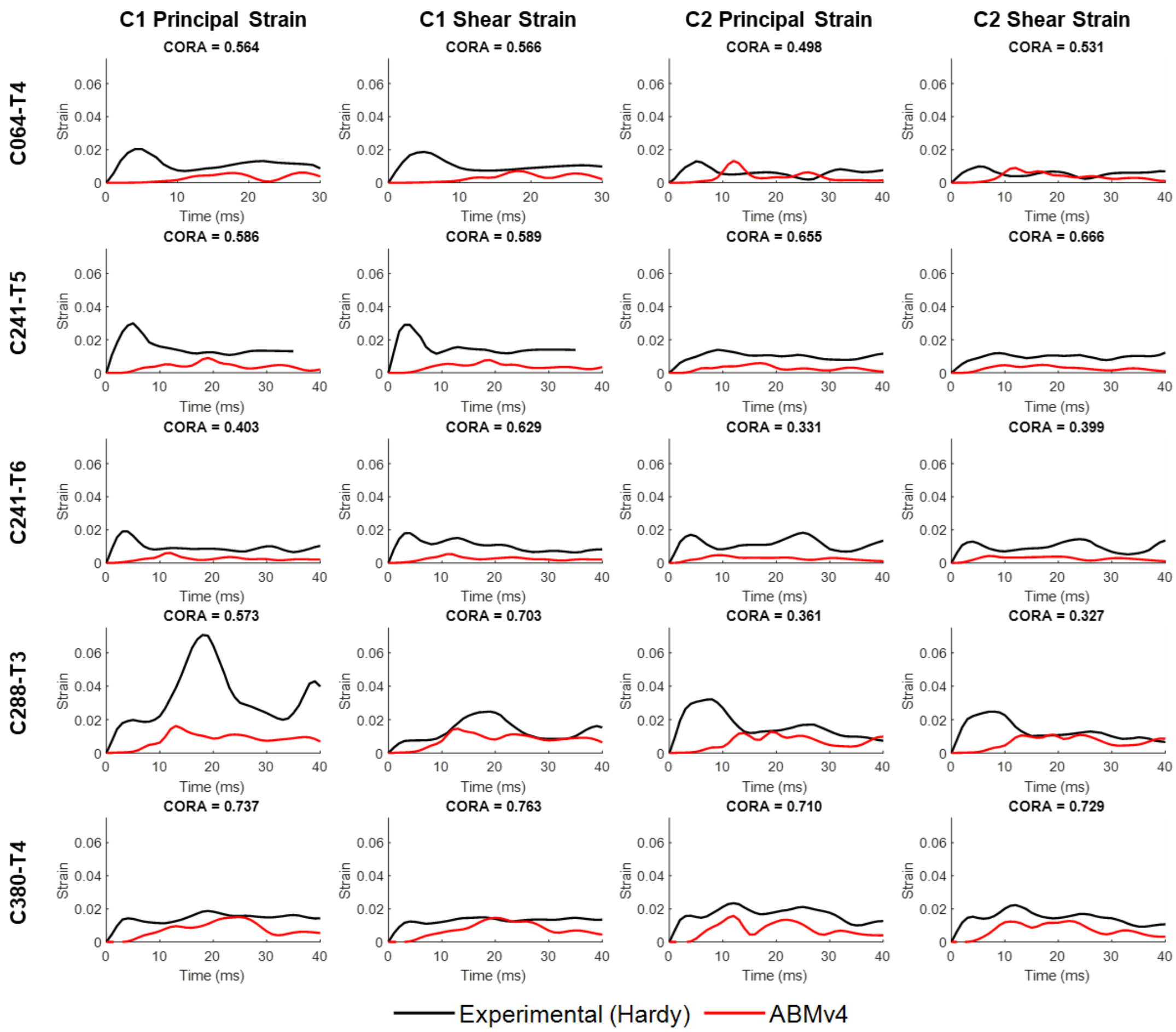

Figure S4. Comparison between experimental and ABMv4 simulated strain responses NDT clusters C1 and C2 for experiments: C064-T4, C241-T5, C241-T6, C288-T3, and C380-T4.

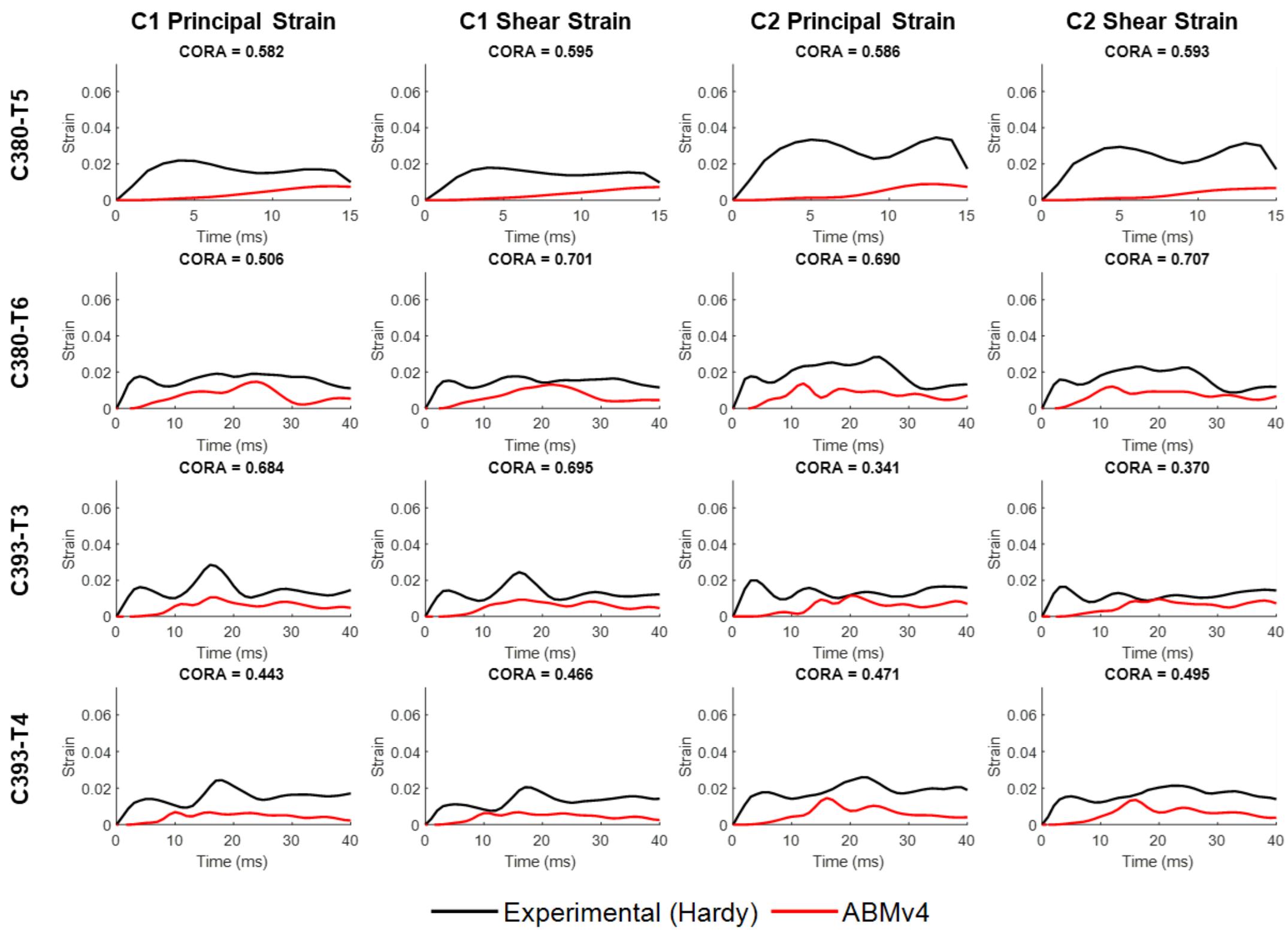

Figure S5. Comparison between experimental and ABMv4 simulated strain responses NDT clusters C1 and C2 for experiments: C380-T5, C380-T6, C393-T3, and C393-T4.

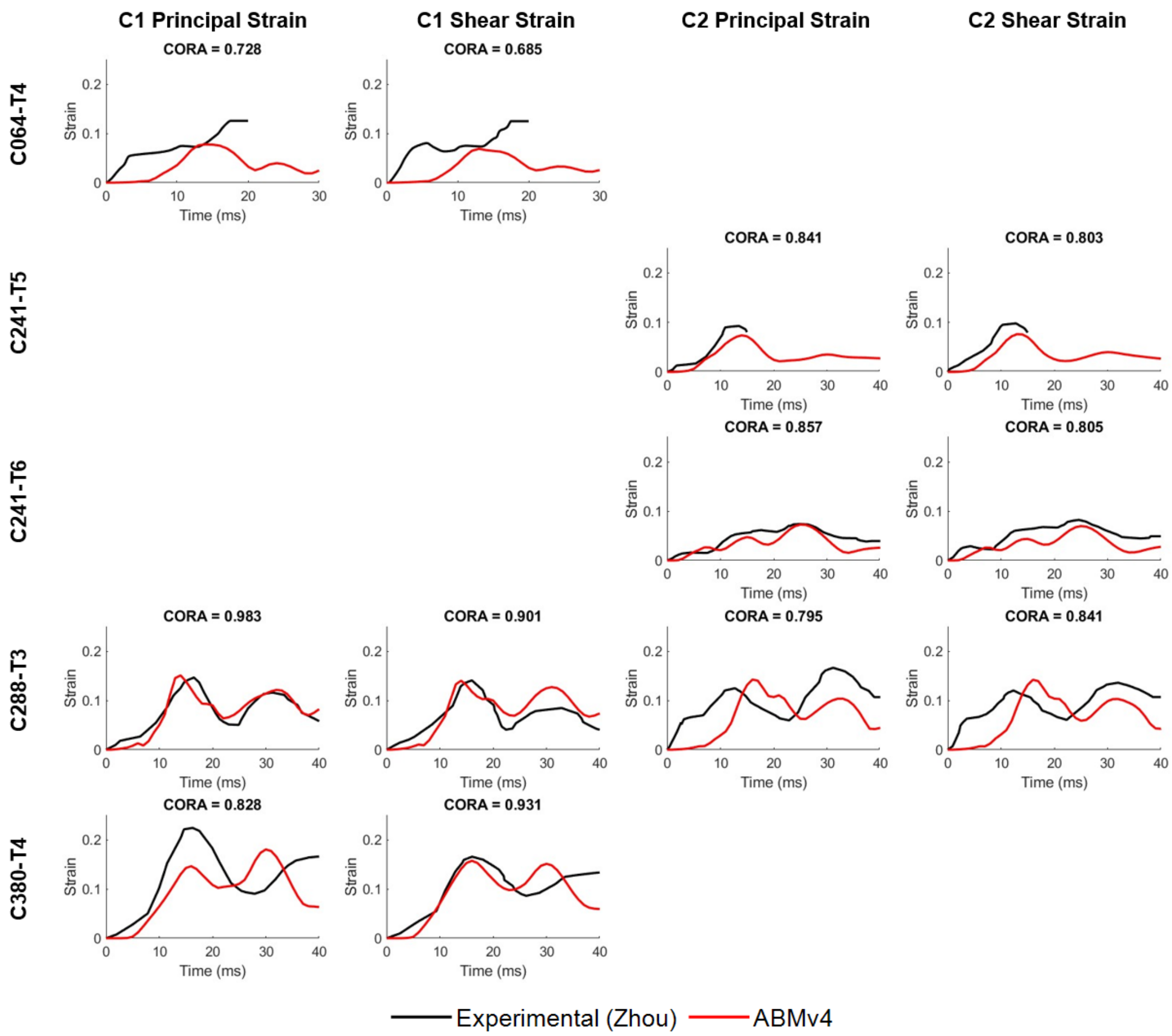

Figure S6. Comparison between revised strain derived by Zhou et al. (2019b) and ABMv4 strain responses at C1 and C2 locations for experiments: C064-T4, C241-T5, C241-T6, C288-T3, and C380-T4.

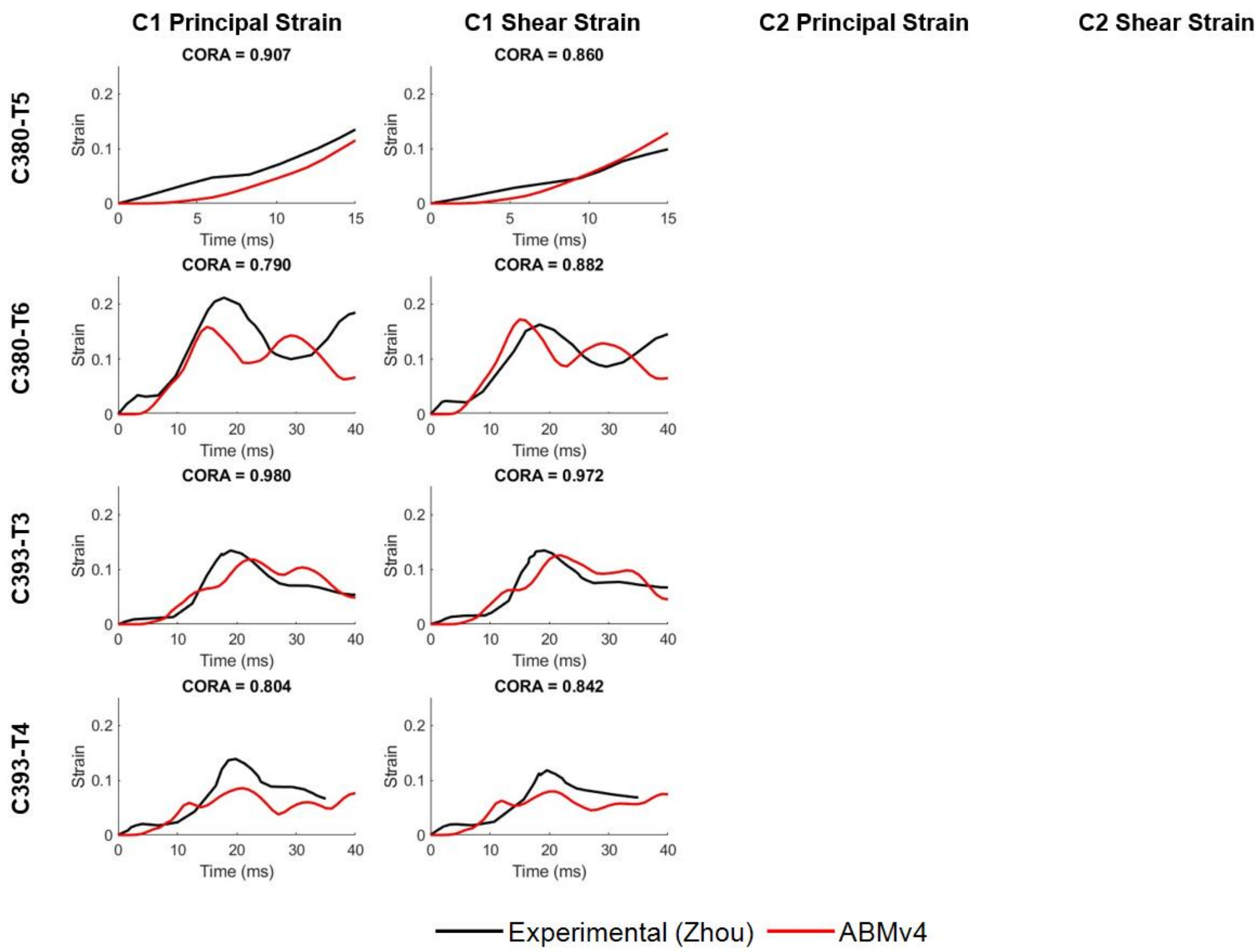

Figure S7. Comparison between revised strain derived by Zhou et al. (2019b) and ABMv4 strain responses at available locations for experiments: C380-T5, C380-T6, C393-T3, and C393-T4.

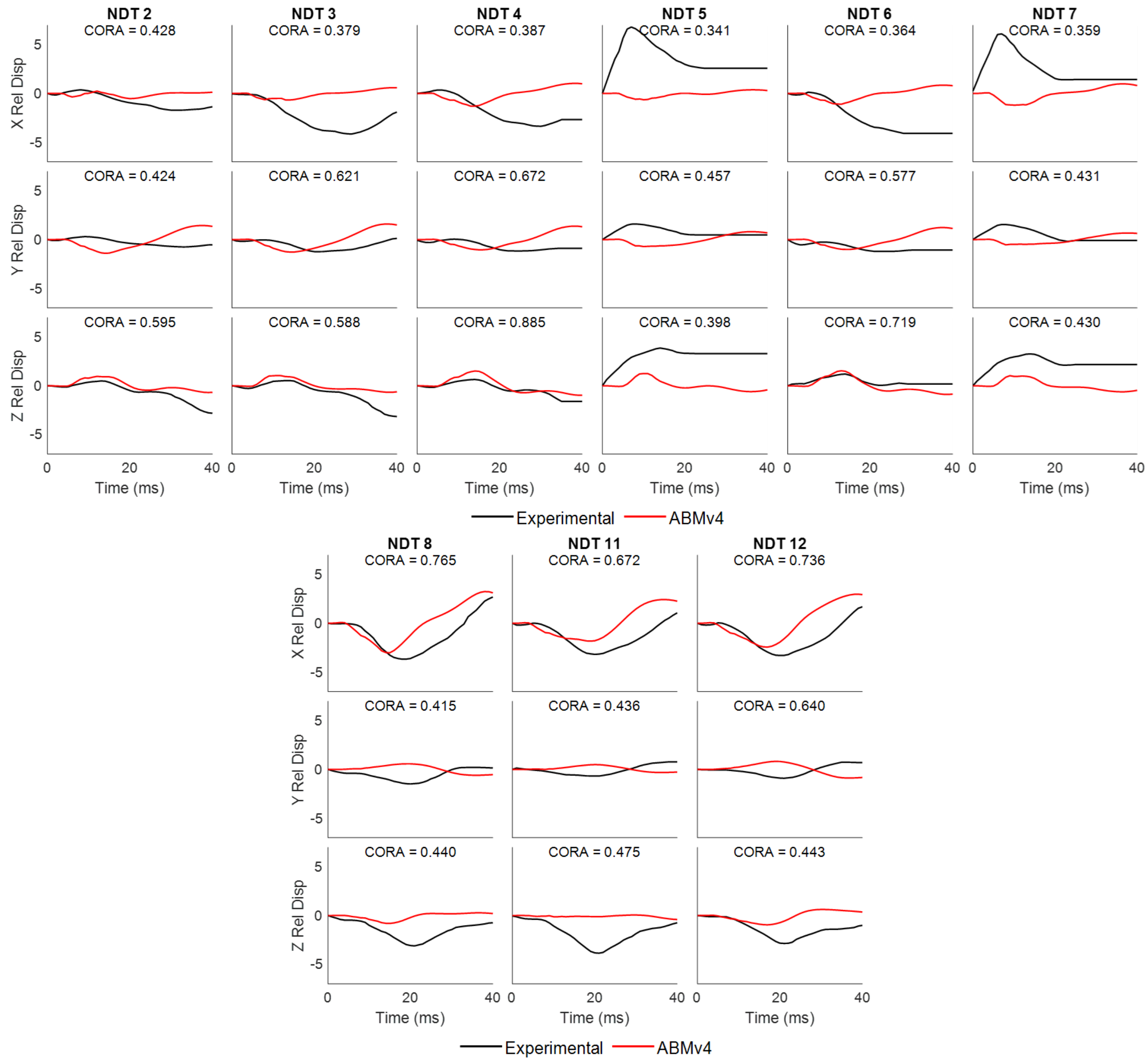

Figure S8. Comparison between experimental and ABMv4 simulated brain motion for experiment C064-T4 at clusters 1 and 2.

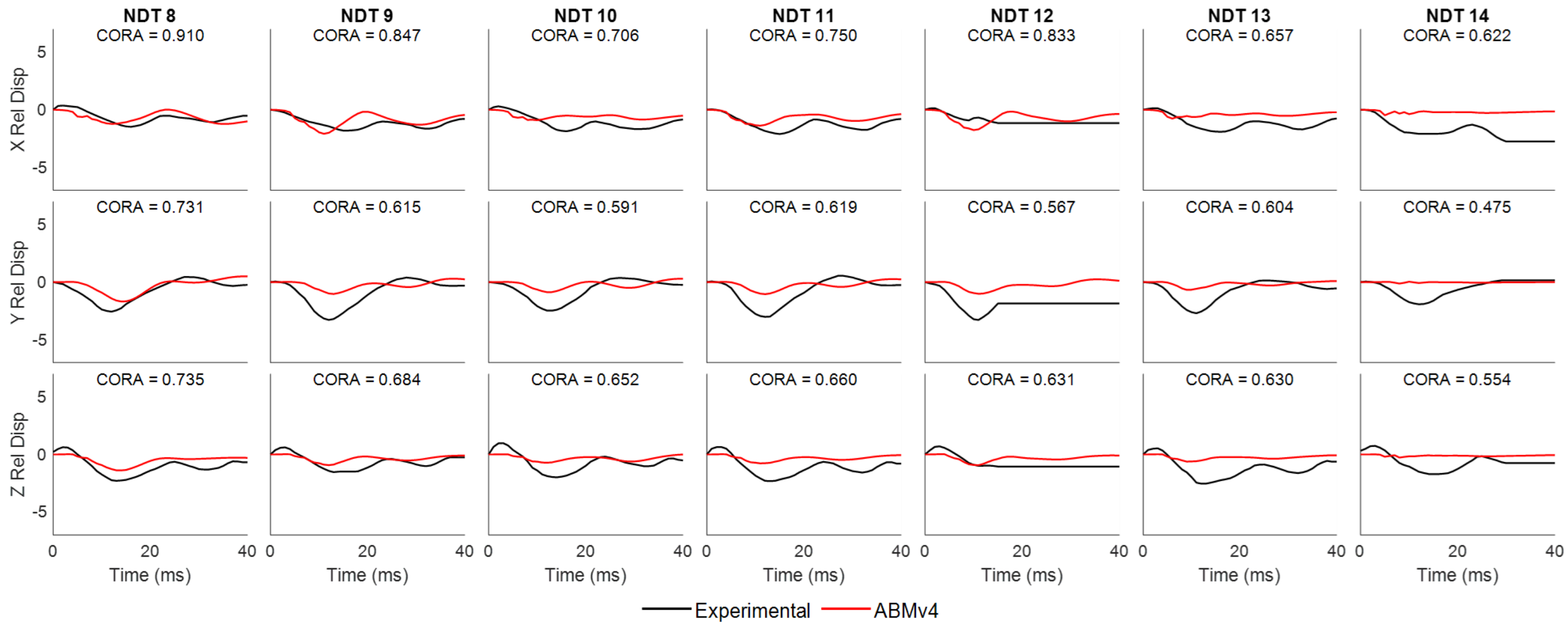

Figure S9. Comparison between experimental and ABMv4 simulated brain motion for experiment C241-T5 at cluster 2.

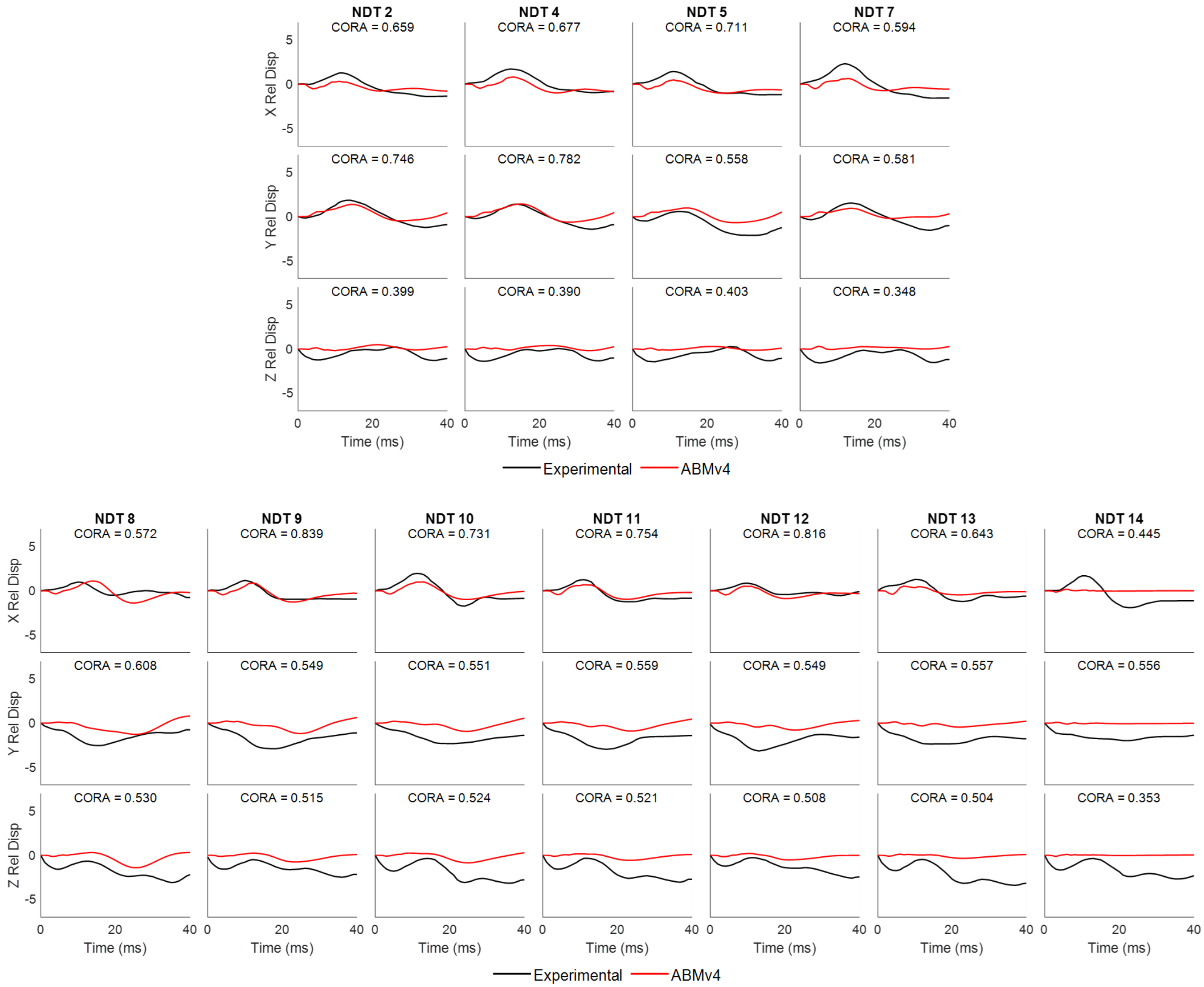

Figure S10. Comparison between experimental and ABMv4 simulated brain motion for experiment C241-T6 at clusters 1 and 2.

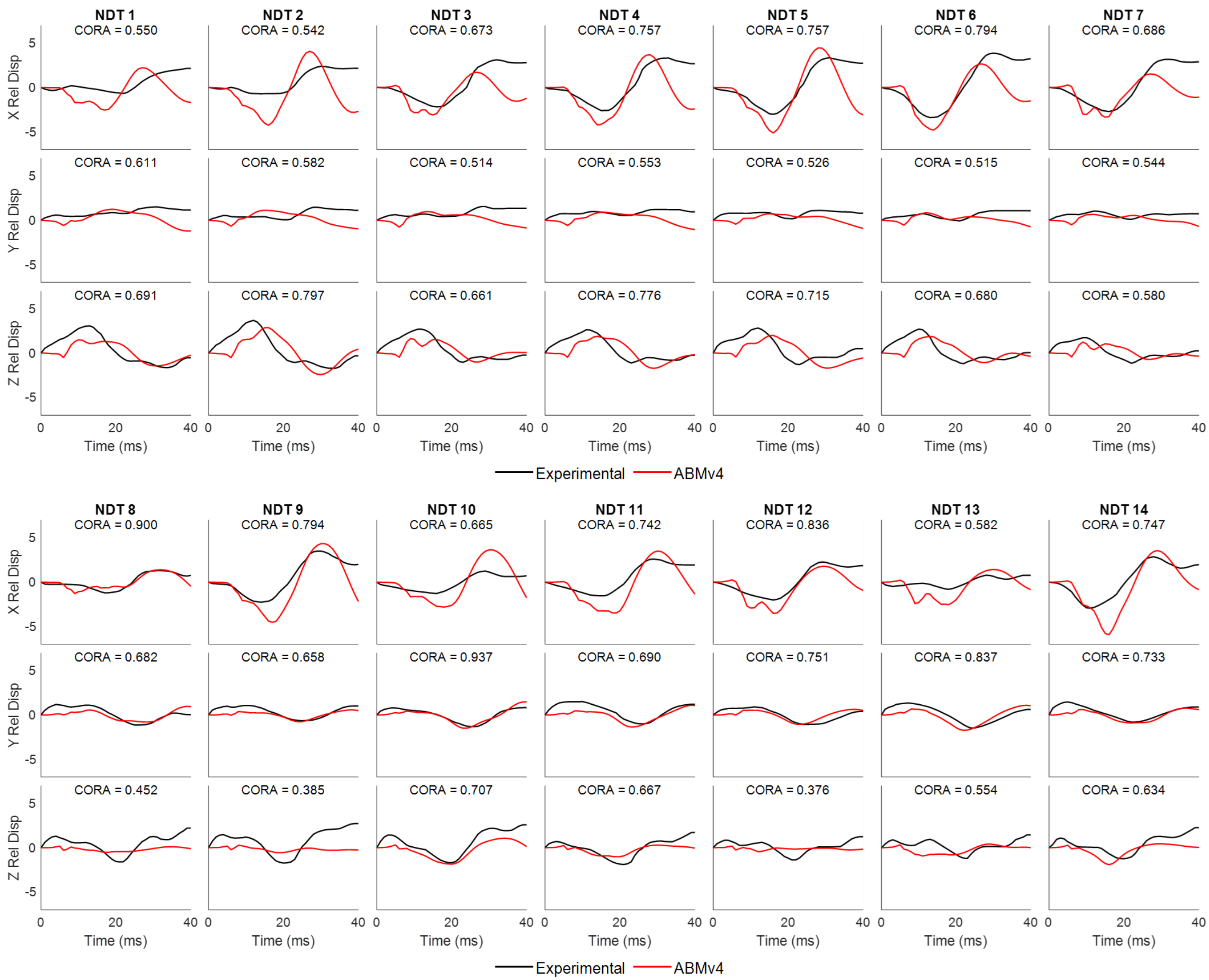

Figure S11. Comparison between experimental and ABMv4 simulated brain motion for experiment C288-T3 at clusters 1 and 2.

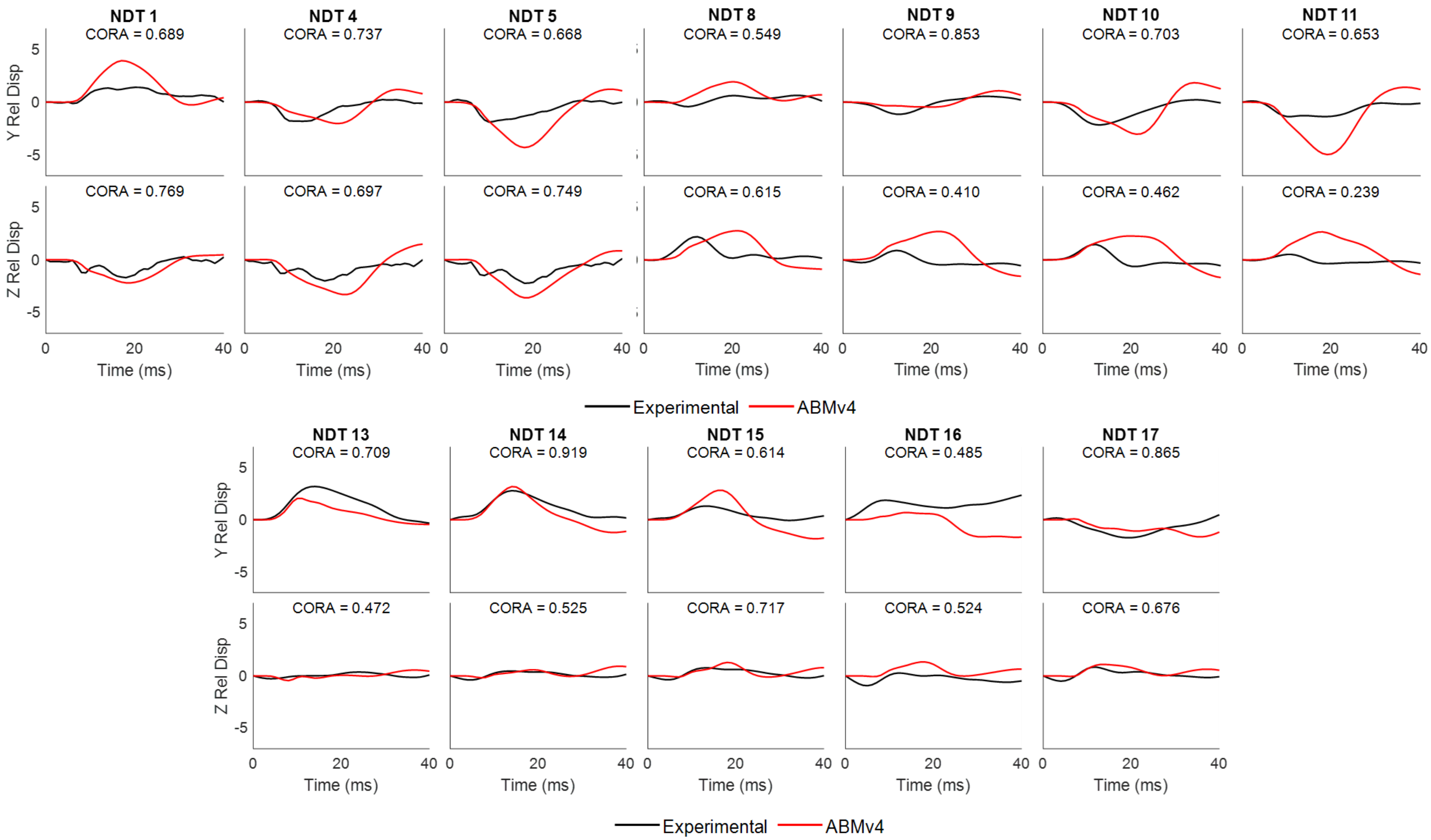

Figure S12. Comparison between experimental and ABMv4 simulated brain motion for experiment C291-T1 at anterior and posterior columns.

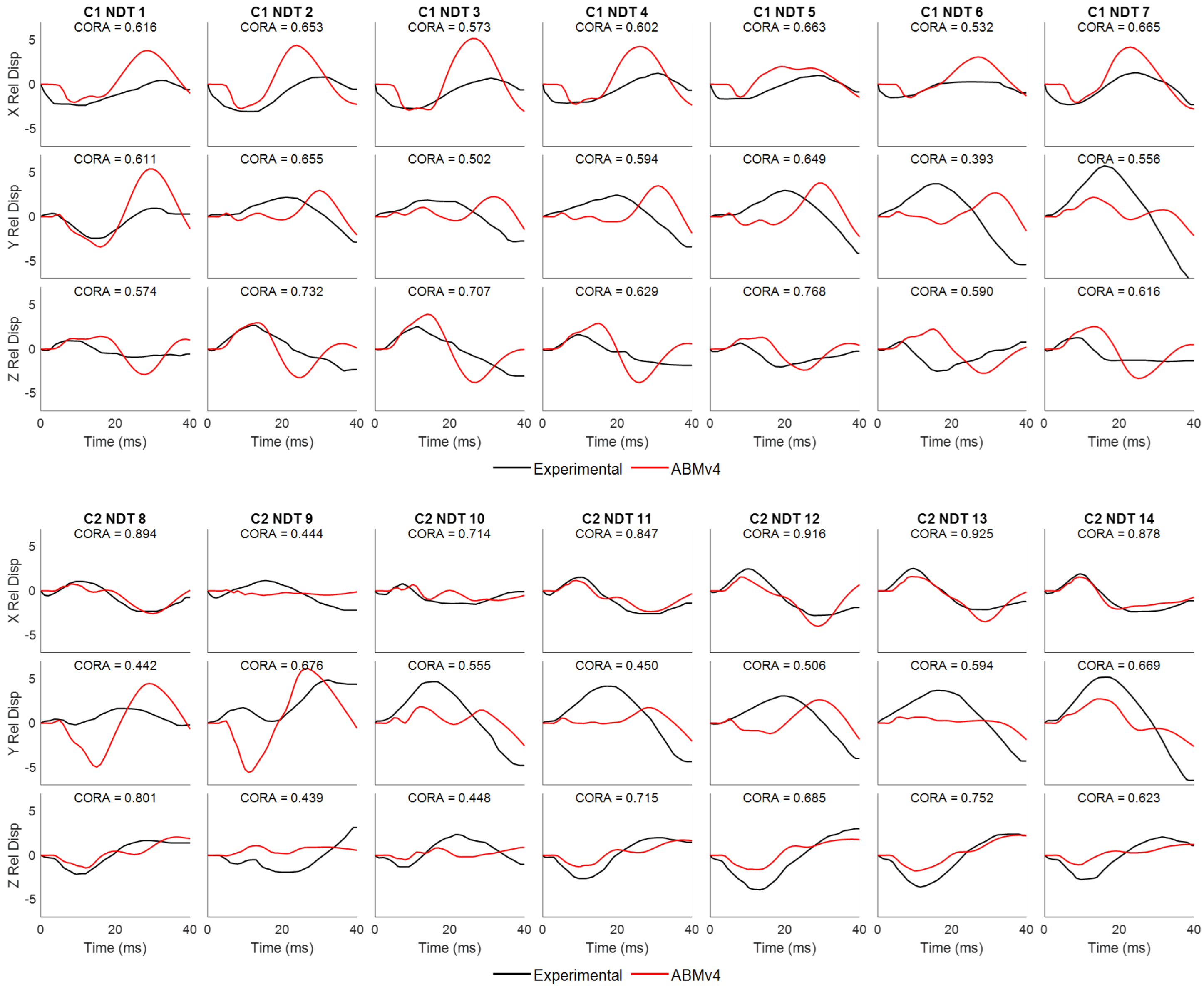

Figure S13. Comparison between experimental and ABMv4 simulated brain motion for experiment C380-T4 at clusters 1 and 2.

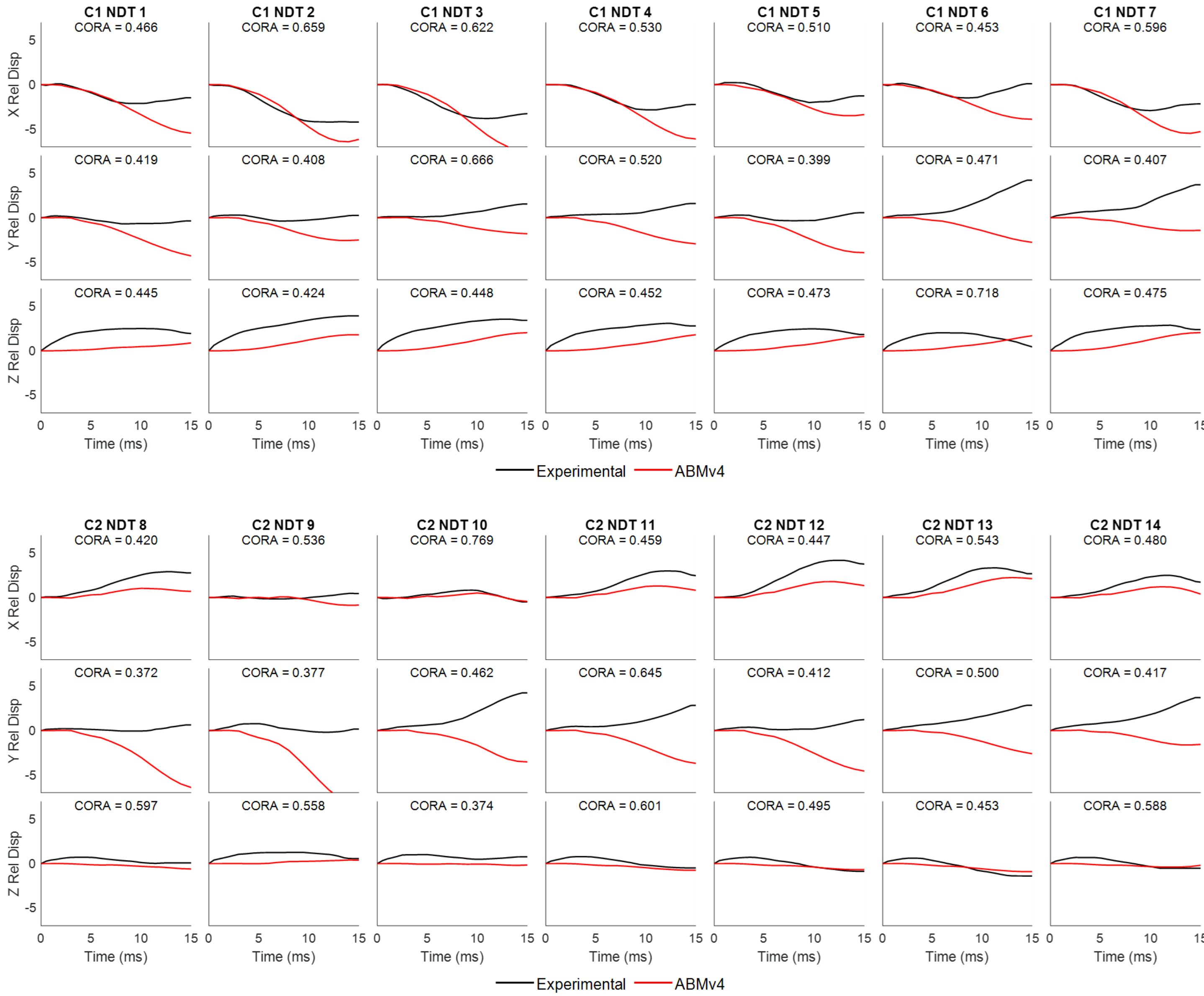

Figure S14. Comparison between experimental and ABMv4 simulated brain motion for experiment C380-T5 at clusters 1 and 2.

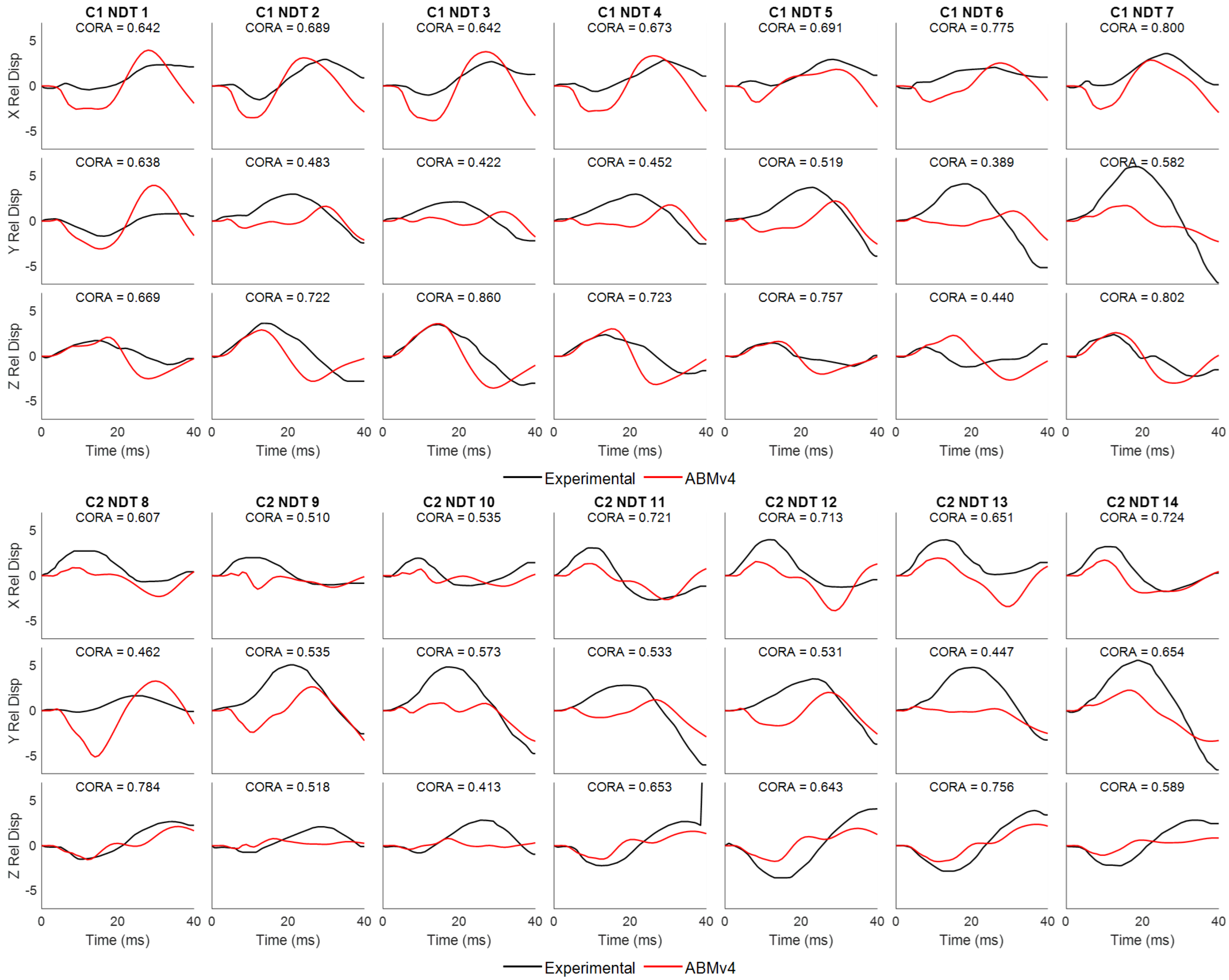

Figure S15. Comparison between experimental and ABMv4 simulated brain motion for experiment C380-T6 at clusters 1 and 2.

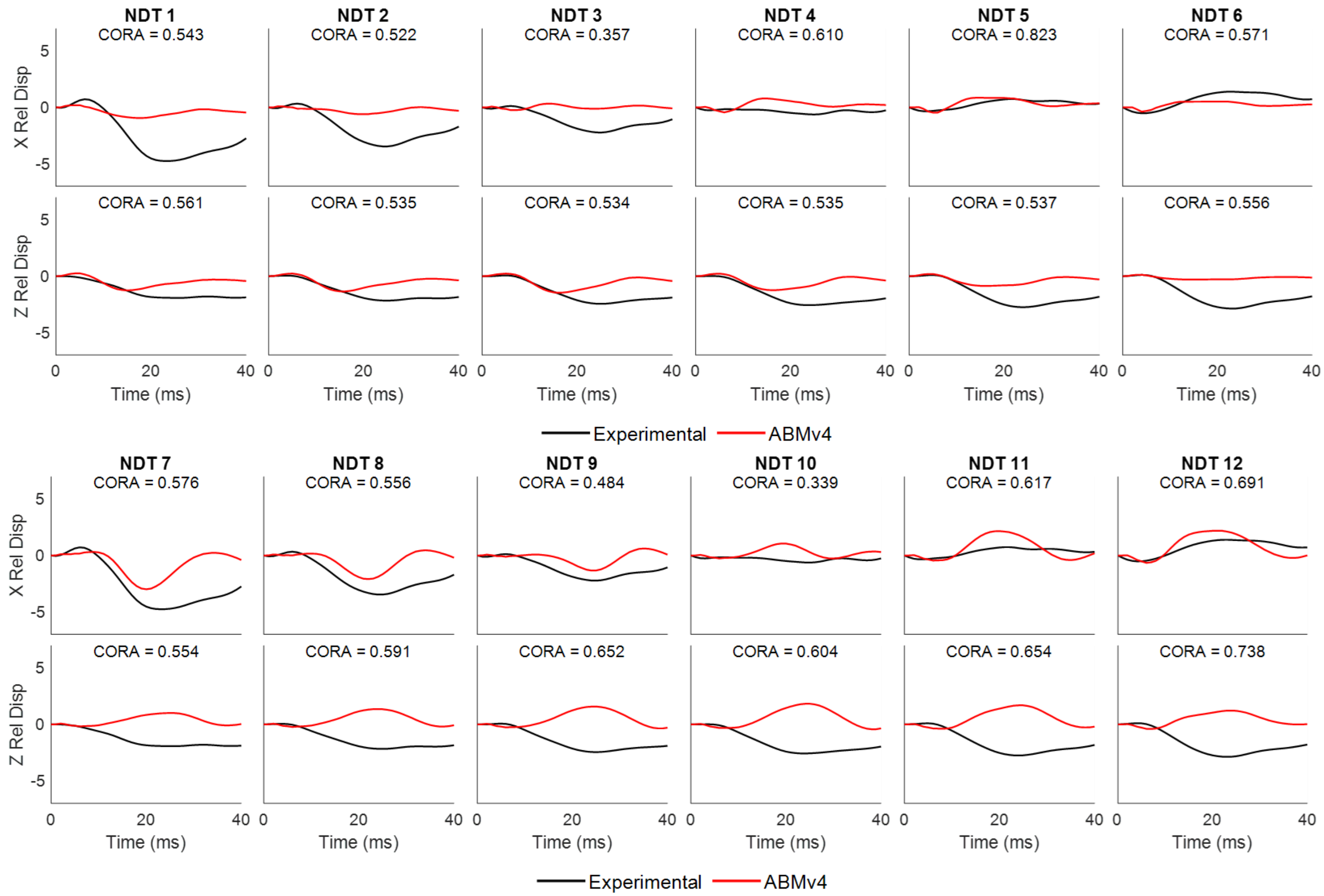

Figure S16. Comparison between experimental and ABMv4 simulated brain motion for experiment C383-T1 at anterior and posterior columns.

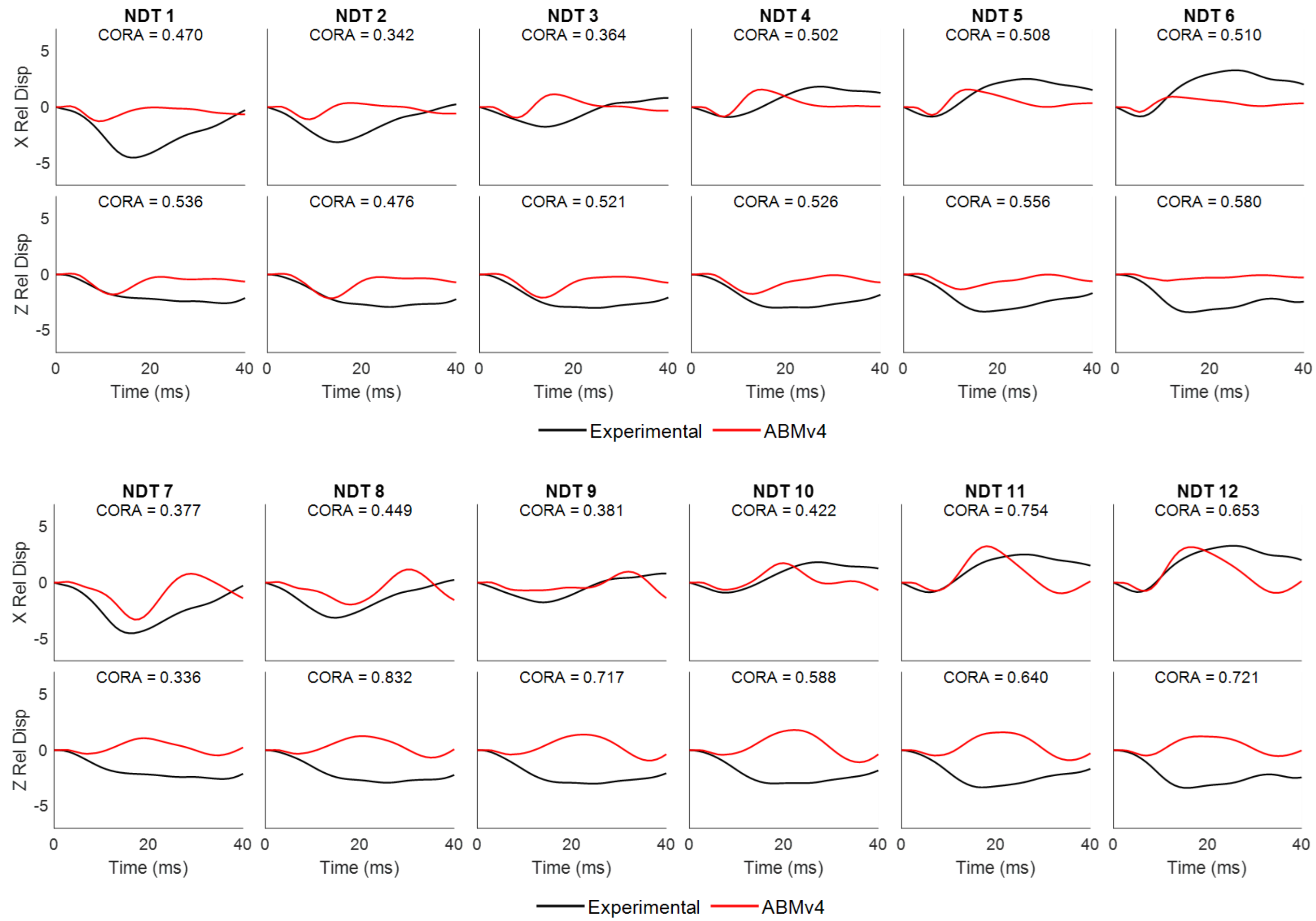

Figure S17. Comparison between experimental and ABMv4 simulated brain motion for experiment C383-T3 at anterior and posterior columns.

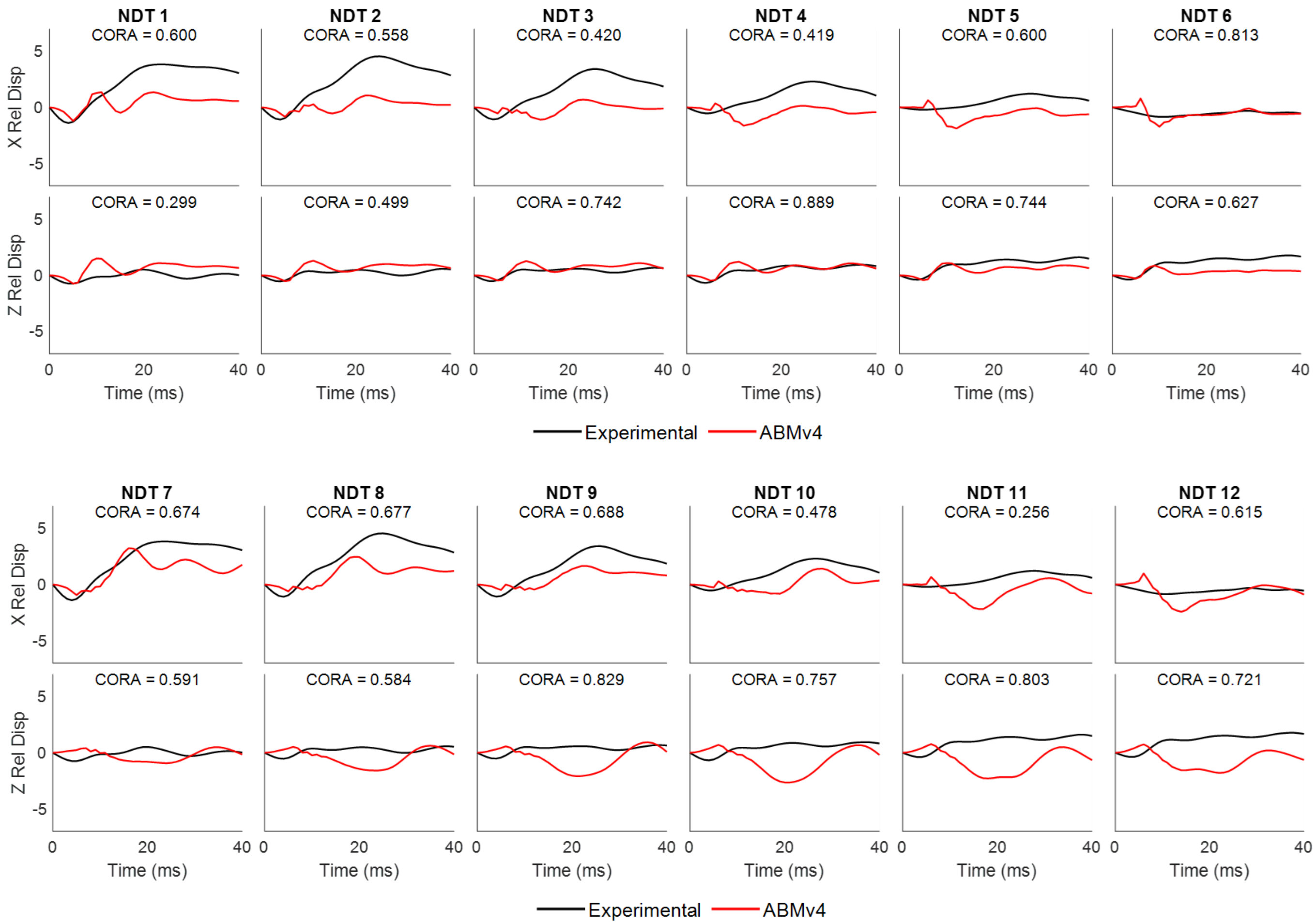

Figure S18. Comparison between experimental and ABMv4 simulated brain motion for experiment C383-T4 at anterior and posterior columns.

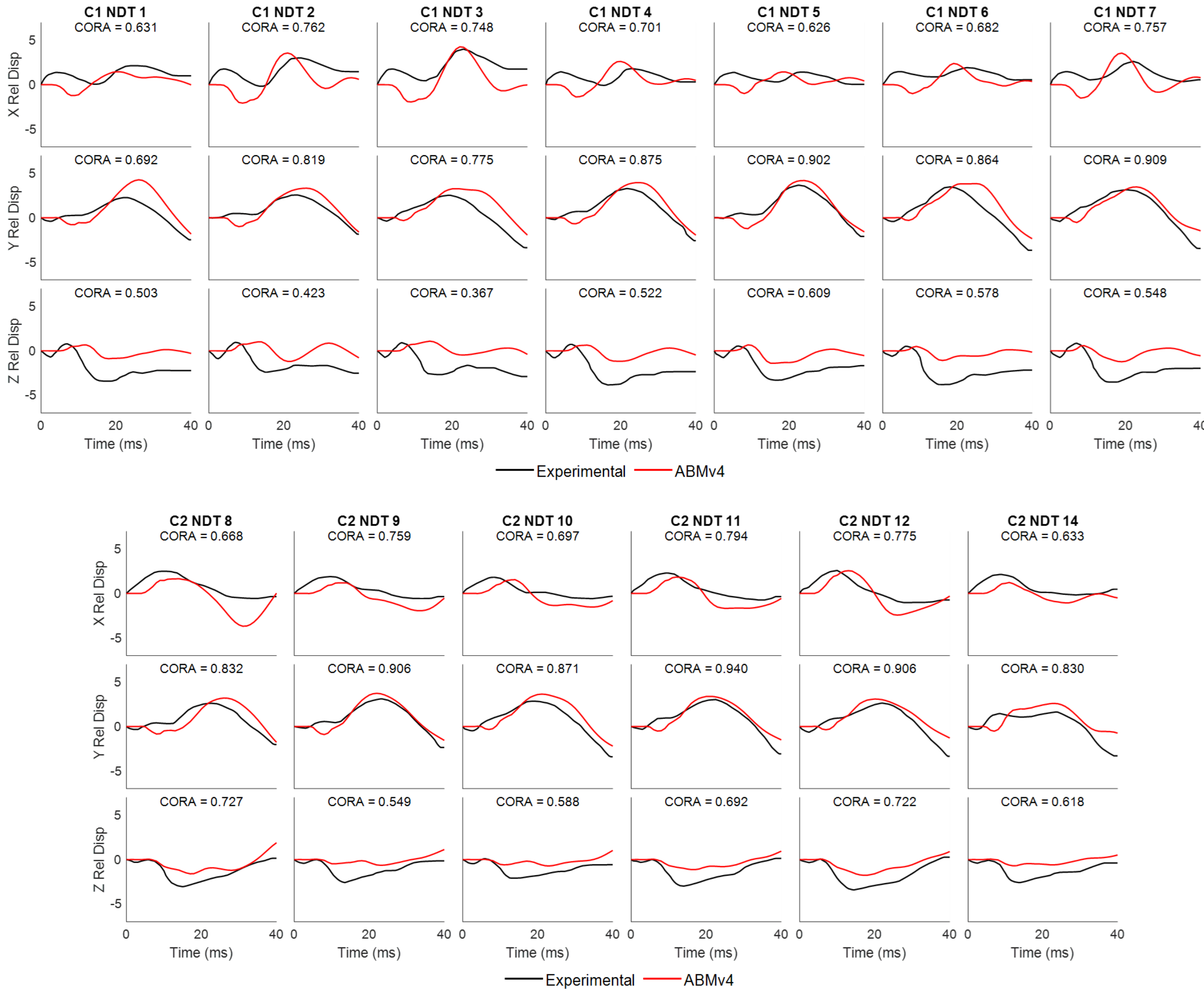

Figure S19. Comparison between experimental and ABMv4 simulated brain motion for experiment C393-T3 at clusters 1 and 2.

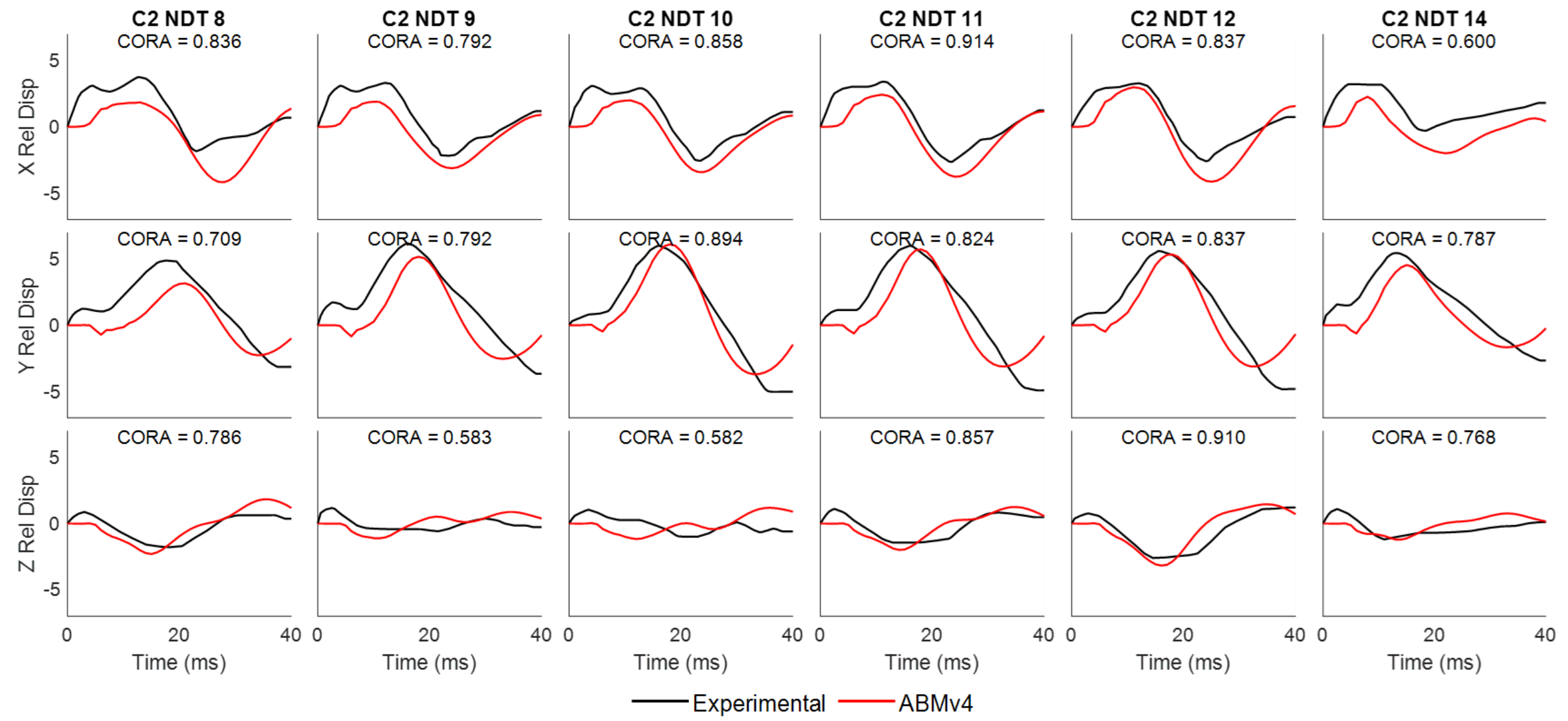

Figure S20. Comparison between experimental and ABMv4 simulated brain motion for experiment C393-T4 at cluster 2.

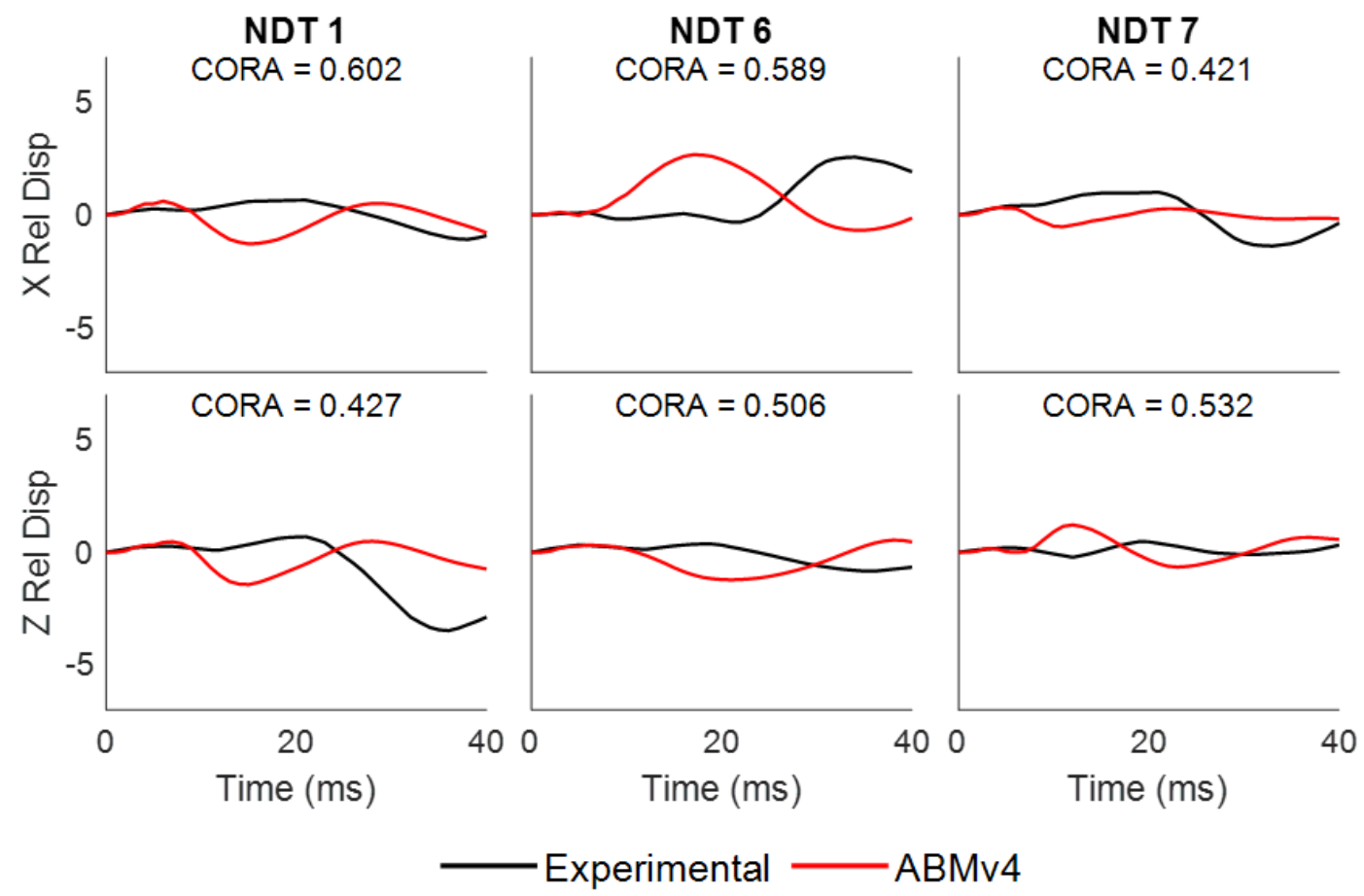

Figure S21. Comparison between experimental and ABMv4 simulated brain motion for experiment C428-T1 at selected NDTs in anterior and posterior columns.

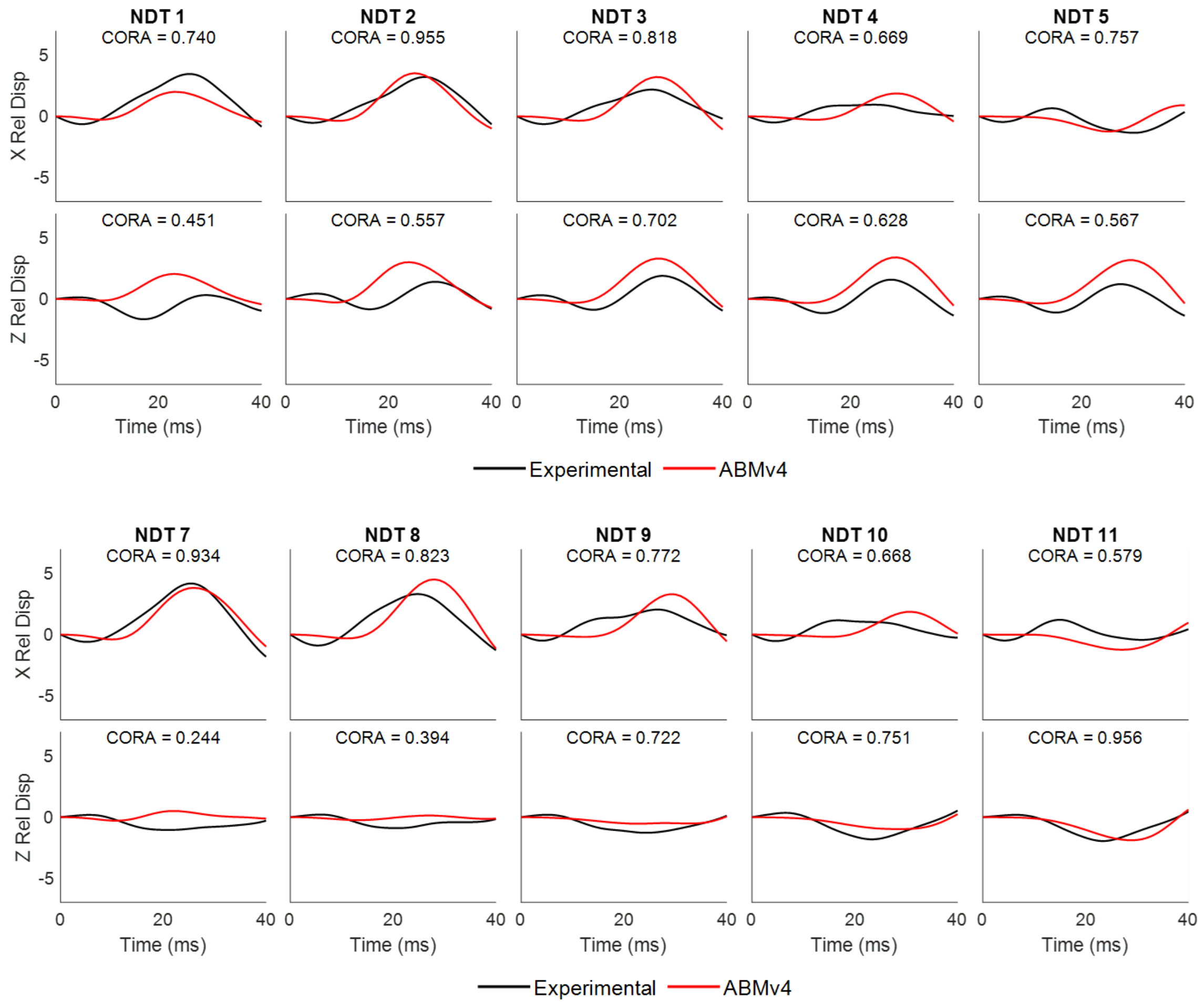

Figure S22. Comparison between experimental and ABMv4 simulated brain motion for experiment C755-T2 at anterior and posterior columns.

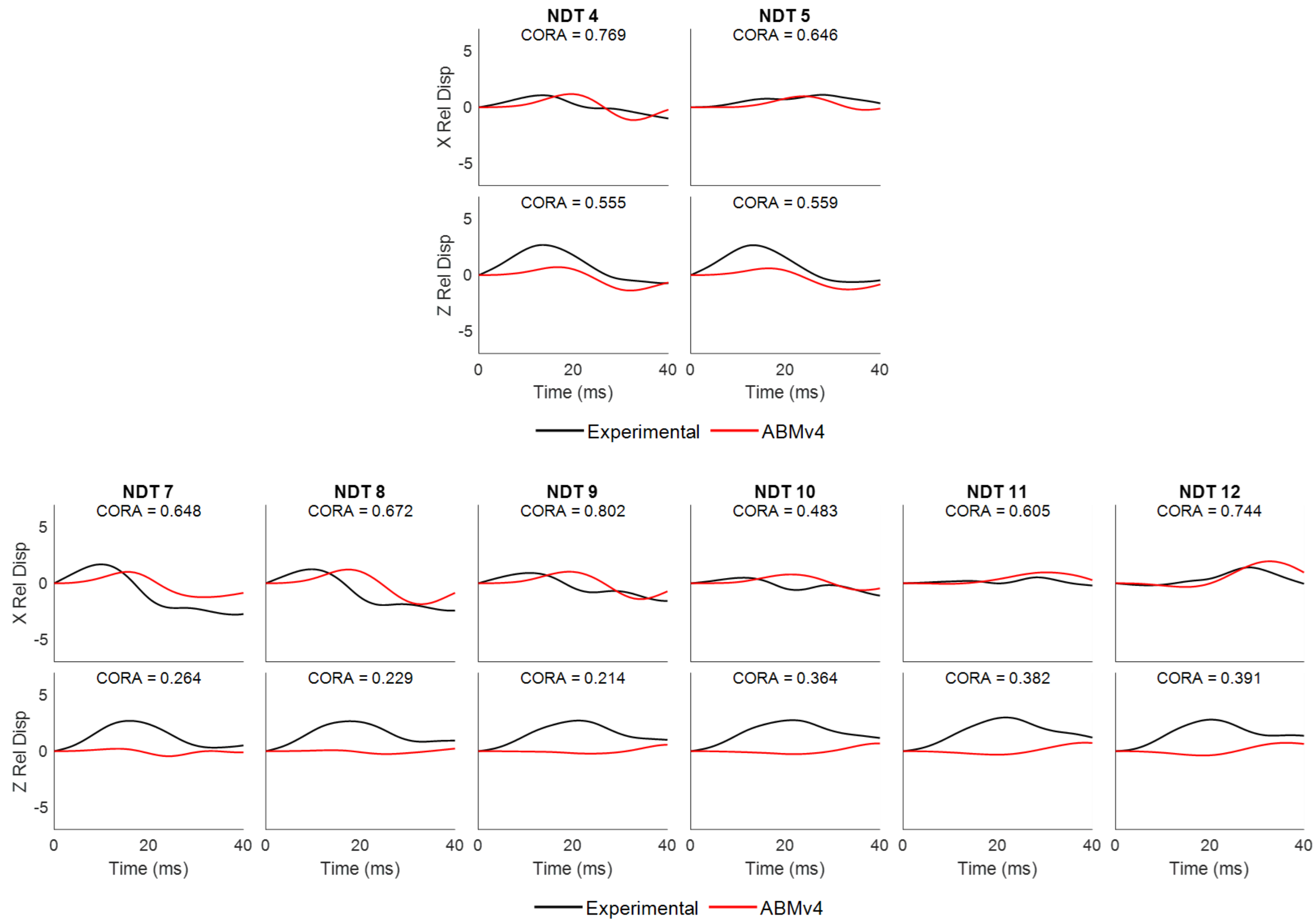

Figure S23. Comparison between experimental and ABMv4 simulated brain motion for experiment C755-T5 at anterior and posterior columns.

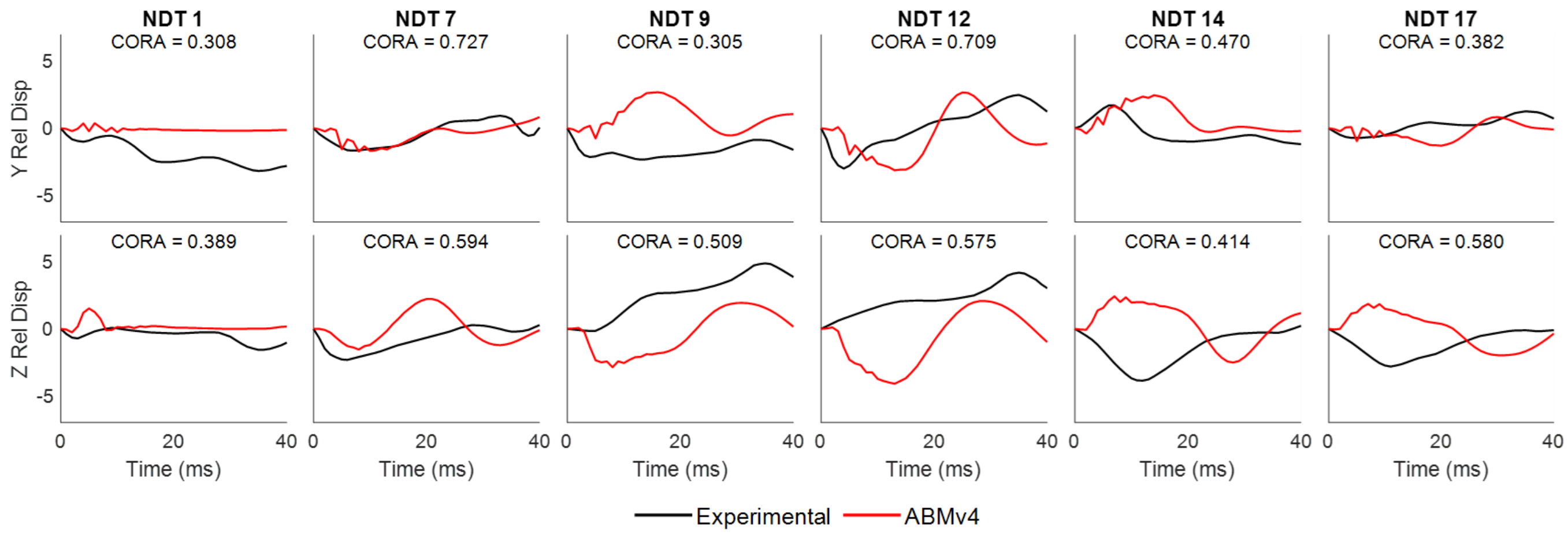

Figure S24. Comparison between experimental and ABMv4 simulated brain motion for experiment C910-T1 at selected NDTs in anterior and posterior columns.

References

Allen JS, Damasio H, Grabowski TJ (2002) Normal neuroanatomical variation in the human brain: An MRI-volumetric study. Am J Phys Anthropol Off Publ Am Assoc Phys Anthropol 118:341–358

Filben TM, Pritchard NS, Miller LE, et al (2022) Characterization of Head Impact Exposure in Women's Collegiate Soccer. J Appl Biomech 38:2–11. https://doi.org/10.1123/jab.2020-0304

Gehre C, Gades H, Wernicke P (2009) Objective rating of signals using test and simulation responses. pp 09–0407

Giordano C, Kleiven S (2016) Development of an Unbiased Validation Protocol to Assess the Biofidelity of Finite Element Head Models used in Prediction of Traumatic Brain Injury. In: 60th Stapp Car Crash Conference. pp 2016-22–0013

Hardy WN, Mason MJ, Foster CD, et al (2007) A study of the response of the human cadaver head to impact. Stapp Car Crash J 51:17

Horgan TJ, Gilchrist MD (2003) The creation of three-dimensional finite element models for simulating head impact biomechanics. IJCrash 8:353–366

Ji S, Zhao W, Ford JC, et al (2015) Group-Wise Evaluation and Comparison of White Matter Fiber Strain and Maximum Principal Strain in Sports-Related Concussion. J Neurotrauma 32:441–454. https://doi.org/10.1089/neu.2013.3268

Marks ME, Holcomb TD, Pritchard NS, et al (2022) Characterizing Exposure to Head Acceleration Events in Youth Football Using an Instrumented Mouthpiece. Ann Biomed Eng 50:1620–1632. https://doi.org/10.1007/s10439-022-03097-7

Mertz H, Irwin A, Melvin J, et al (1989) Size, weight and biomechanical impact response requirements for adult size small female and large male dummies. SAE Technical Paper

Miller LE, Urban JE, Stitzel JD (2016) Development and validation of an atlas-based finite element brain model. Biomech Model Mechanobiol 15:1201–1214. https://doi.org/10.1007/s10237-015-0754-1

Rich AM, Filben TM, Miller LE, et al (2019) Development, Validation and Pilot Field Deployment of a Custom Mouthpiece for Head Impact Measurement. Ann Biomed Eng 47:2109–2121. https://doi.org/10.1007/s10439-019-02313-1

Toga AW, Mazziotta JC (2002) Brain Mapping: The Methods. Academic press

Zhou Z, Li X, Kleiven S, Hardy WN (2019) Brain Strain from Motion of Sparse Markers. Stapp Car Crash J 63:1–27. https://doi.org/10.4271/2019-22-0001
